# Supplementary material for: A conformal piezoelectric microsystem for demographic-adaptive and calibration-free cuffless blood pressure monitoring
Source: Nat Commun. 2025 Dec 9;17:439. doi: 10.1038/s41467-025-67118-4 (PMC12799606; doi:10.1038/s41467-025-67118-4)
Supplement: Supplementary file 1 — Supplementary Information [file 41467_2025_67118_MOESM1_ESM.pdf]

Supplementary Information for

A Conformal Piezoelectric Microsystem for Demographic-Adaptive and Calibration-Free Cuffless Blood Pressure Monitoring

Cunman Liang[1,2,3,#], Zhou Jiang[1,2,4,#], Shirong Qiu[1,#], Lei Zhao[1,2,4], Xinxin Mao[1], Xiao Yang[2], Yuanting Zhang[1,5], Ni Zhao[1,2]*

[1]Department of Electronic Engineering, The Chinese University of Hong Kong, Hong Kong, China,
[2]Hong Kong Centre for Cerebro-cardiovascular Health Engineering (COCHE), Hong Kong, China,
[3]Key Laboratory of Mechanism Theory and Equipment Design of Ministry of Education, Tianjin University, Tianjin, China,
[4]Department of Biomedical Engineering, City University of Hong Kong, Hong Kong, China,
[5]AICARE Bay Lab at Guangdong Medical University, Dong-Guan, China,

[#] These authors contributed equally: Cunman Liang, Zhou Jiang, Shirong Qiu

* Corresponding author. Email: nzhao@ee.cuhk.edu.hk

Supplementary Note 1: Comparison of cuffless BP monitoring technologies

In standard practice, blood pressure (BP) assessment traditionally involves employing a sphygmomanometer equipped with an inflatable cuff. BP values are obtained by inflating a cuff to a pressure higher than BP and then recording the value at which the intra-arterial pressure is equal to the cuff pressure. This method can only record temporally discrete BP value, which results in the inability to record the patient's physiological status in a timely manner and may even result in the omission of vital signs [1-3]. At present, invasive methods (arterial catheterization) are used to obtain continuous BP in clinical applications, which requires placing a sensor into a blood vessel through a catheter to record BP values in real time[4]. This method can get most accurate BP values, but this will cause harm to the patient [5,6].

Cuffless BP monitoring technologies enable non-invasive, continuous BP monitoring, providing real-time insights into cardiovascular dynamics, enabling early hypertension detection and supporting personalized healthcare. Therefore, it is very important to study non-invasive devices for continuous BP monitoring. Common cuffless BP monitoring devices can be categorized into four types: pressure-based, optical, electrical, and acoustic devices. The working principles, limitations, and advantages of these devices are listed in the following Tab. 1. For these types of BP monitoring devices, calibration is still necessary. Efforts have been made to reduce the frequency of calibration, such as making electronic BP device ultra-thin or configuring the ultrasound sensor in a small-spaced array format. However, these devices based on a single sensor still cannot achieve calibration-free BP monitoring due to limited measurement data.

Table 1. Comparison between different kinds of BP monitoring devices.

Device type	Working principle	Limitation	Advantage	Calibration	Reference
Pressure-based	Direct conversion: Pressure transmission	Need supporting structure	High stability	Frequent	[7, 8]
Optical	Indirect conversion: PTT/PAT method	Limited penetration depth (<8 mm) Easy disturbed	Convenience, and efficiency	Frequent	[9, 10]
Electrical	Indirect conversion: PTT/PAT method	Easy disturbed by electrical noise	Low positioning requirement	Ultra-thin structure, reducing calibration times	[11, 12]
Acoustic	Indirect conversion: Wall-tracking, resonance sonomanometry	Relative high positioning requirement, Require intimate contact without any gap.	Large penetration depth	Large area array with small pitch, reducing calibration times	[13, 14]

Supplementary Note 2: Finite element analysis (FEA)

The mechanical deformations and strain distributions of the PWF sensor with and without a cavity were obtained by FEA, using the commercial software ANSYS (version 19.0). The minimum mesh size of the finite element model (see Fig. S2) is 1.5 μm, and the total number of elements is greater than 220,000, which can ensure the accuracy of the calculation. The Young's modulus and Poisson's ratio used in the analysis were 83 GPa and 0.37 for Ag; 2.5 GPa and 0.34 for PI; 2 GPa and 0.39 for PVDF; 1.8 MPa and 0.49 for PDMS; 70 kPa and 0.49 for the soft elastomer (Ecoflex 00-30, Smooth-on). In the simulation, the lower surface of the device was fixed and a constant displacement load was applied at the center of the end of the piezoelectric beam, which is consistent with the experiment.

Supplementary Note 3: Fabrication procedures for conformal and stretchable piezoelectric microsystem

Preparing electrodes of ultrasound sensor

1. Clean glass slide with acetone, iso-Propyl alcohol (IPA), and deionized water;
2. Spin coat PDMS (1:10) at 3000 rpm on glass slide;
3. Bake at 70 ˚C for 1 h;
4. Transfer Cu/polyimide(PI) (18μm/12.5μm) film onto PDMS-coated glass slide;
5. Cut outline of electrodes with laser cutter (LPKF Protolaser U4), peel off extra Cu/ PI film;

Preparing substrate

6. Spin coat dextran (10 wt%, dissolved in water) at 1500 rpm on cleaned glass slide;
7. Bake at 80 ˚C for 1 min, 180 ˚C for 30 min;
8. Spin coat Ecoflex 00-30 at 1500 rpm on the dextran-coated glass slide;
9. Bake at 70 ˚C for 1 h;

Ultrasound sensor

10. Transfer bottom electrode on Ecoflex substrate by water-soluble tape;
11. Bond ultrasound transducers and Cu cube on the bottom electrode by solder paste;
11. Transfer top electrode on PDMS stamp by water-soluble tape;
12. Align and bond the top electrode with the transducers and Cu cube;
13. Remove the PDMS stamp (designated as **DEVICE 1**);

PWF sensor

14. Laminate PVDF film (TE Connectivity, 28 μm) onto PDMS-coated glass slide;
15. Cut outline of PWF sensor with laser cutter (LPKF Protolaser U4), peel off extra PVDF film;
16. Transfer PWF sensor on another Ecoflex substrate by water-soluble tape;
17. Pour PDMS (1:10) onto a 3D-printed cavity mold, cure at 70 ℃ for 1 h;
18. Detach PDMS from mold as PDMS cavity;
19. Bond PDMS cavity onto PWF sensor by uncured Ecoflex (designated as **DEVICE 2**);

Dual-model CSPM

20. Place the top surface of **DEVICE 2** onto uncured Ecoflex;
21. Align and place **DEVICE 2** with **DEVICE 1** through markers using a microscopic visual system;
22. Bake at 70 ˚C for 1 h;
23. Pour Ecoflex into the gap between **DEVICE 1 and DEVICE 2**;
24. Bake at 70 ˚C for 1 h;
25. Remove glass slide in warm water to form the dual-model CSPM;
26. Blade coat Silbione (RT Gel 4717, Bluestar) on the surface of dual-model CSPM;

Supplementary note 4: General cuffless BP derivations

Consider a general relationship between pressure and cross-sectional area (*A*) in a vascular segment [15], which can be described by

$$P = \theta + \gamma e^{\beta \cdot A} \tag{1}$$

Where *P* is the blood pressure, β and γ are pressure-independent constants. θ is related to intrinsic properties of an artery to remain patent at very low pressures and γ is a scaling factor for the y-intercept. *A* is the cross-sectional area of the vascular segment. β is the beta stiffness, which is expressed as

$$\beta = \frac{\ln[(SBP - \theta)/(DBP - \theta)]}{\Delta A} \tag{2}$$

where *SBP* is systolic blood pressure, *DBP* is diastolic blood pressure, ΔA is vascular cross-sectional area difference between systolic and diastolic phase and can be measured by ultrasound sensor.

Then the BP components can be expressed as

$$SBP = PSR \cdot DBP + \theta(1 - PSR) \tag{3}$$

where *PSR* is pulse stiffening ratio.

$$PSR = e^{\beta \cdot \Delta A} \tag{4}$$

Hence, based on this, the beta stiffness can be derived.

$$\beta = \frac{\ln(PSR)}{\Delta A} \tag{5}$$

Since *PSR* is equivalent to ambulatory vascular stiffness index (*AASI*) and *AASI* is positively proportional to pulse wave velocity[16]. Hence,

$$\beta = \frac{\ln\left(\frac{1}{1 - AASI}\right)}{\Delta A} = \frac{\ln\left(\frac{1}{1 - a \cdot PWV + b}\right)}{\Delta A} \tag{6}$$

where *a* and *b* are constants and associated with the relationship of *PSR* and *PWV* in population characteristic.

On the other hand, based on Equation (1), the variation of area is expressed as the variation of the BP,

$$\frac{dP}{dA} = (P - \theta) \cdot \beta \tag{7}$$

Furthermore, the relation between PWV and BP is expressed as follows.

$$PWV^2 = \frac{A}{\rho}\frac{dP}{dA} \tag{8}$$

where ρ is the blood density.

Hence, BP can be expressed as the combination of vascular characteristic parameters.

$$P = \frac{PWV^2}{A \cdot \beta / \rho} + \theta \tag{9}$$

Since θ is related to intrinsic properties of an artery to remain patent at a very low pressure, we assume it is negligible by implicitly decomposed in the dynamic term of those parameters in equation (9). Let

$\mu=\beta/\rho$ is the blood linear density with unit (kg/m). Therefore, μ can be estimated by substituting the PWV and diameters, i.e.,

$$\hat{\mu}=\frac{\beta}{\rho}=\frac{\ln\left(1-\frac{1}{a\cdot PWV+b}\right)}{\Delta A}/\rho \tag{10}$$

where ρ ranges from 1043 to 1060 kg/m^3 (1.043-1.060 g/mL) which variation is smaller than β, see Supplementary table 1.

Notably, the linear blood density can be reliably estimated due to the relative variation of beta stiffness index ($\Delta\beta/\beta$=~0.82)[15] is significantly larger than that of blood density ($\Delta\rho/\rho$=~0.016)[17]. The beta stiffness or μ induced BP is expressed as follows.

$$P=\frac{PWV^2}{A\cdot\beta/\rho}+\theta=\frac{PWV^2}{A}\cdot\mu+\theta \tag{11}$$

where PWV is the pulse wave velocity; A is the cross-sectional area of arteries. Note that all these parameters are subject-specific and could not be negligible in BP measurement. The BP in equation (11) is the rate of change of pulse velocity per unit volume, pushing the blood in the lumen of a blood vessel, in the direction of propagation of the pulse wave.

Supplementary note 5: Time-decay compensation strategy for continuous BP tracking

In above BP measurement, the sensor patch is required to capture parameters without any slippage. However, in practice, sensor patch slippage on skin is unavoidable due to high degree of the body motion and the sensor patch being too slight. The similarity of the local region sensor parameters can compensate for this measurement error. Here, we propose a slippage-free time-decay compensation strategy. The BP pulse time decay calculation is expressed as follows.

$$\tau_{BP} = \frac{T_c}{\beta\left[\frac{D_s^*}{D_d}\cdot\left(1-e^{-\frac{T_c}{\tau_D}}\right)\right]} \tag{12}$$

where τ_{BP} is the pulse time decay of blood pressure; T_c is the heartbeat cycle; τ_D is the pulse time decay of the measured pulse waveforms from ultrasound sensors; D_s^* is the systolic blood vessel diameter; D_d is the diastolic blood vessel diameter; β is the beta stiffness.

The τ_{BP} captures the trend of BP fluctuation that compensates the characterized cuffless BP in equation (11). Finally, the continuous BP (BP_c) can be expressed as follows.

$$BP_c = c \cdot \tau_{BP} / \tau_{BP0} + d + P \tag{13}$$

where c and d are preset constants. τ_{BP0} is the initial time decay corresponding to the characterized cuffless BP measurement. P

Supplementary Figs. 35 and 36 and Fig. 5 b&c show a comparison of various blood pressure models in continuous blood pressure waveforms tracking. A significant enhancement in the precision and consistency of blood pressure measurements is validated by Supplementary Fig. 37, which demonstrates heightened accuracy (mean difference μ = -0.18 mmHg, standard deviation σ = 2.15 mmHg) in blood pressure assessment when employing the proposed BP algorithm, clearly evidencing a notable reduction in measurement inaccuracies.

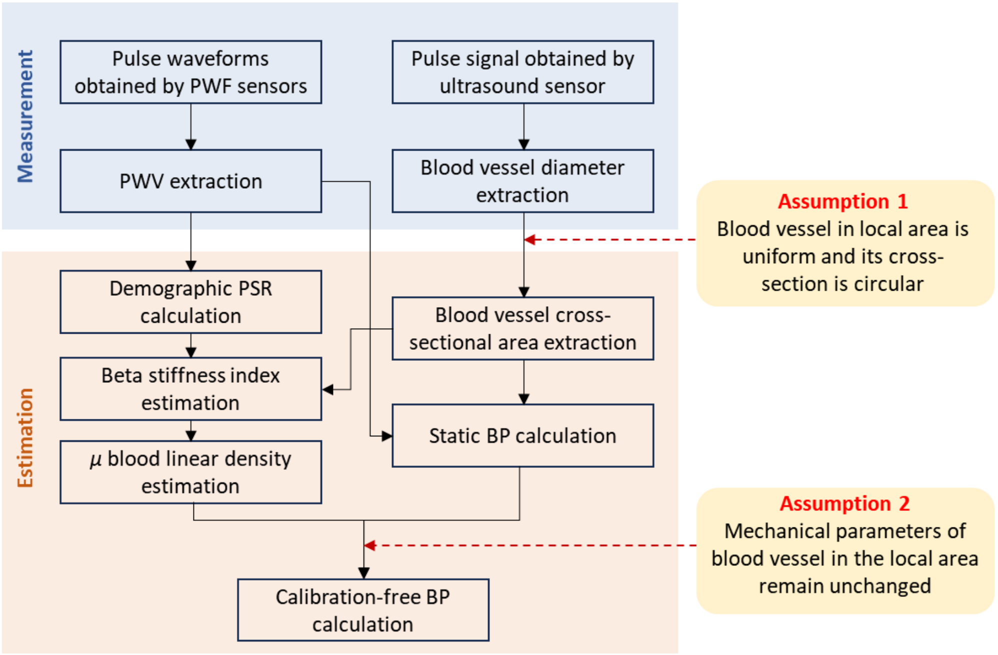

Supplementary Fig. 1. Flowchart of calibration-free BP calculation.

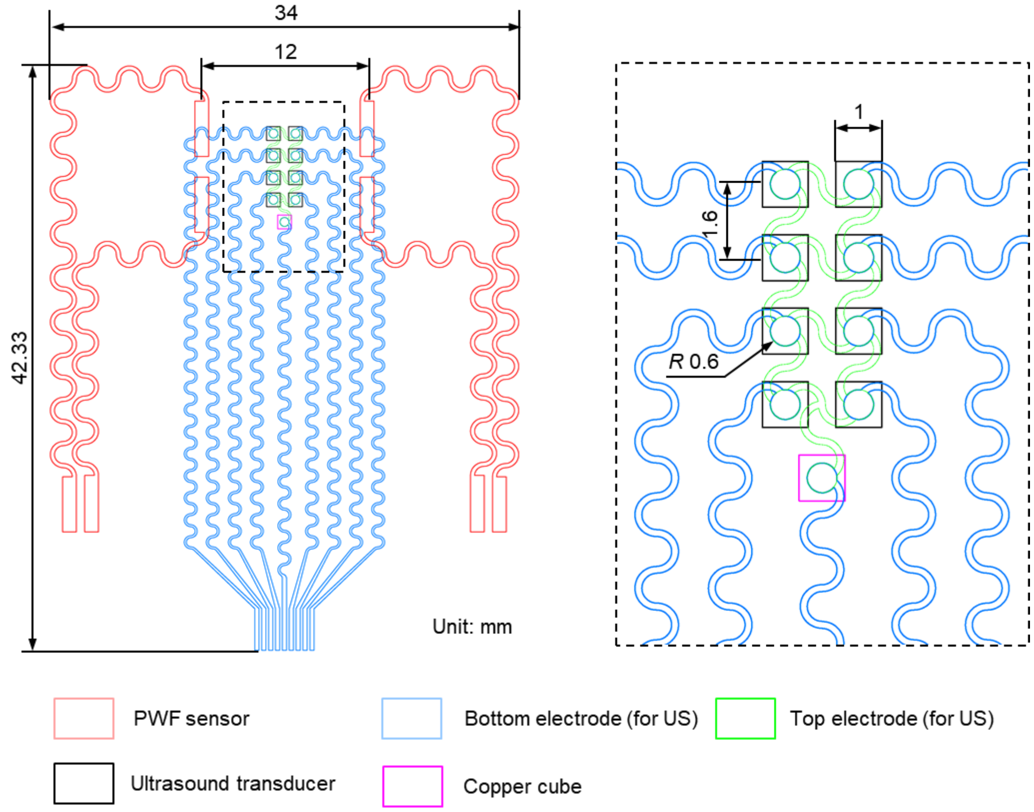

Supplementary Fig. 2. Layout of the CSPM with main dimensional parameters.

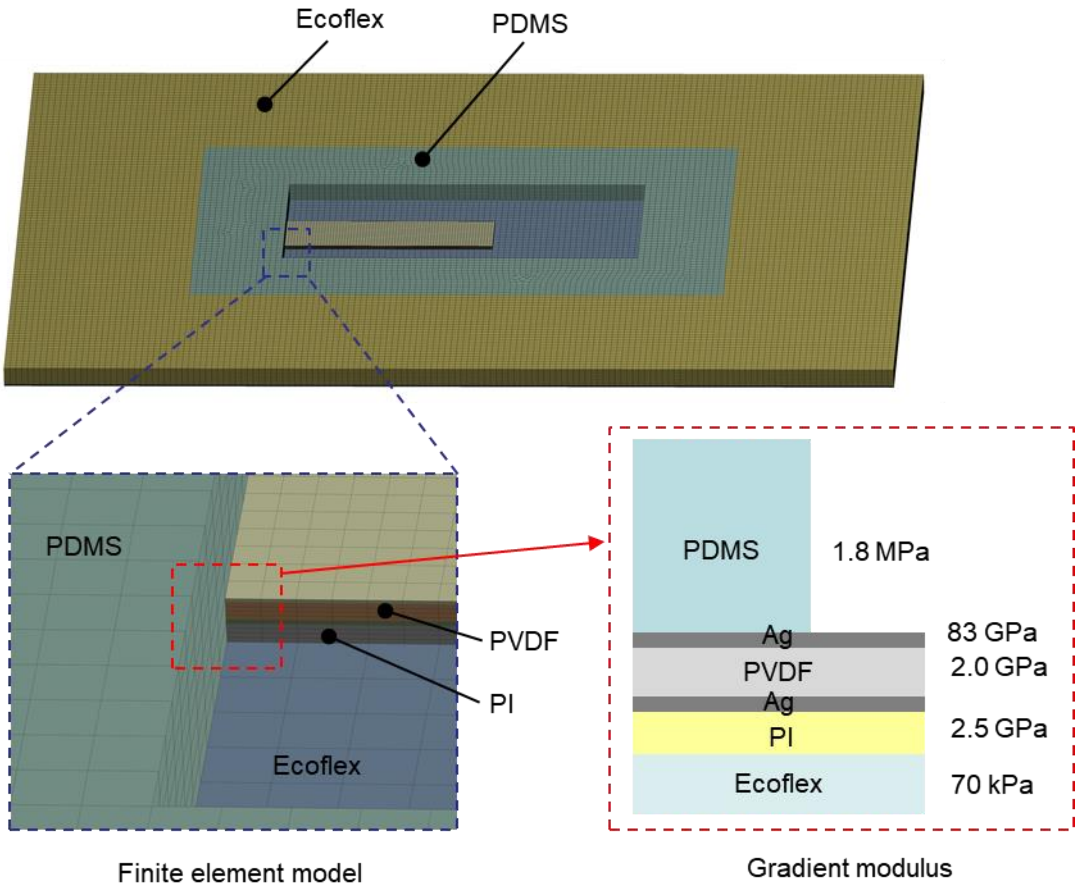

Supplementary Fig. 3. Finite element model of PWF sensor with gradient modulus.

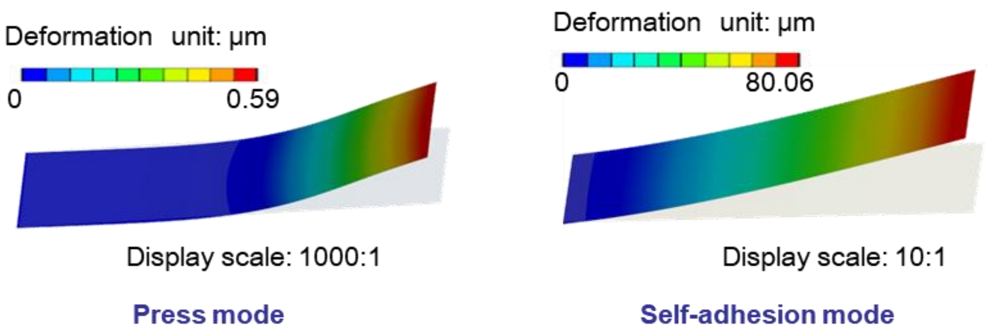

Supplementary Fig. 4. Deformation distribution of press mode and self-adhesion mode PWF sensors by finite element analysis, showing that the self-adhesion mode PWF sensor has larger deformation than the press mode PWF sensor under the same load.

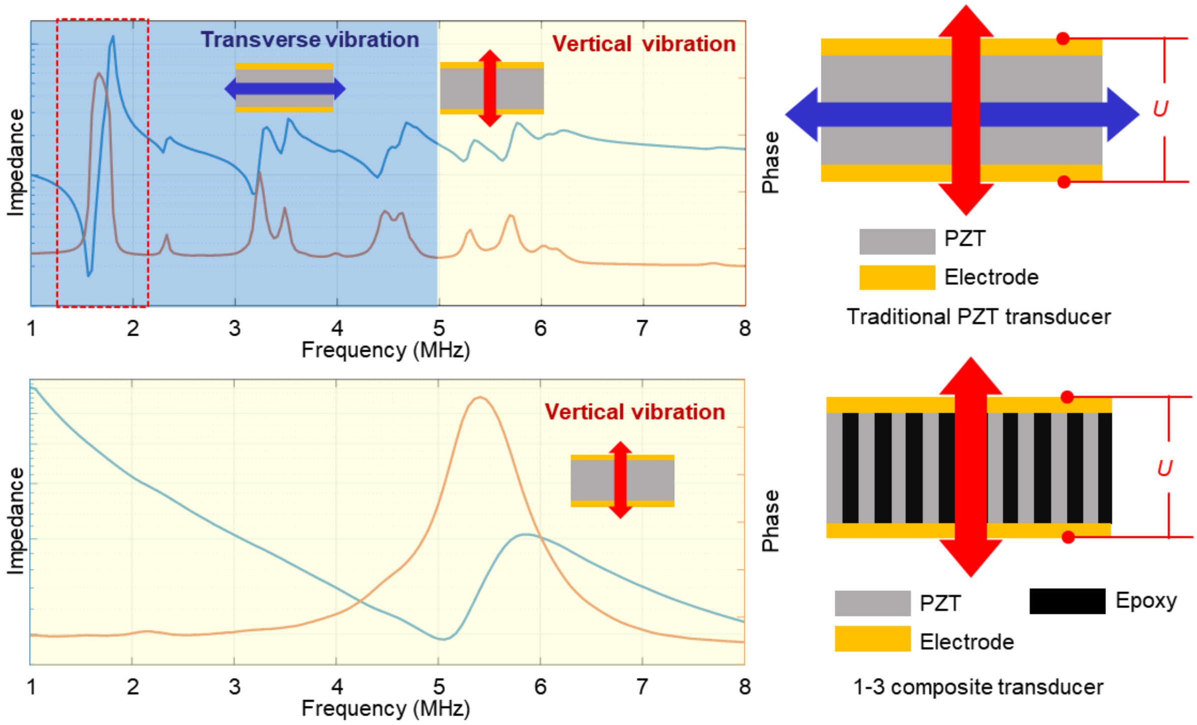

Supplementary Fig. 5. Comparison of vibration modes between traditional PZT transducer and 1-3 composite transducer.

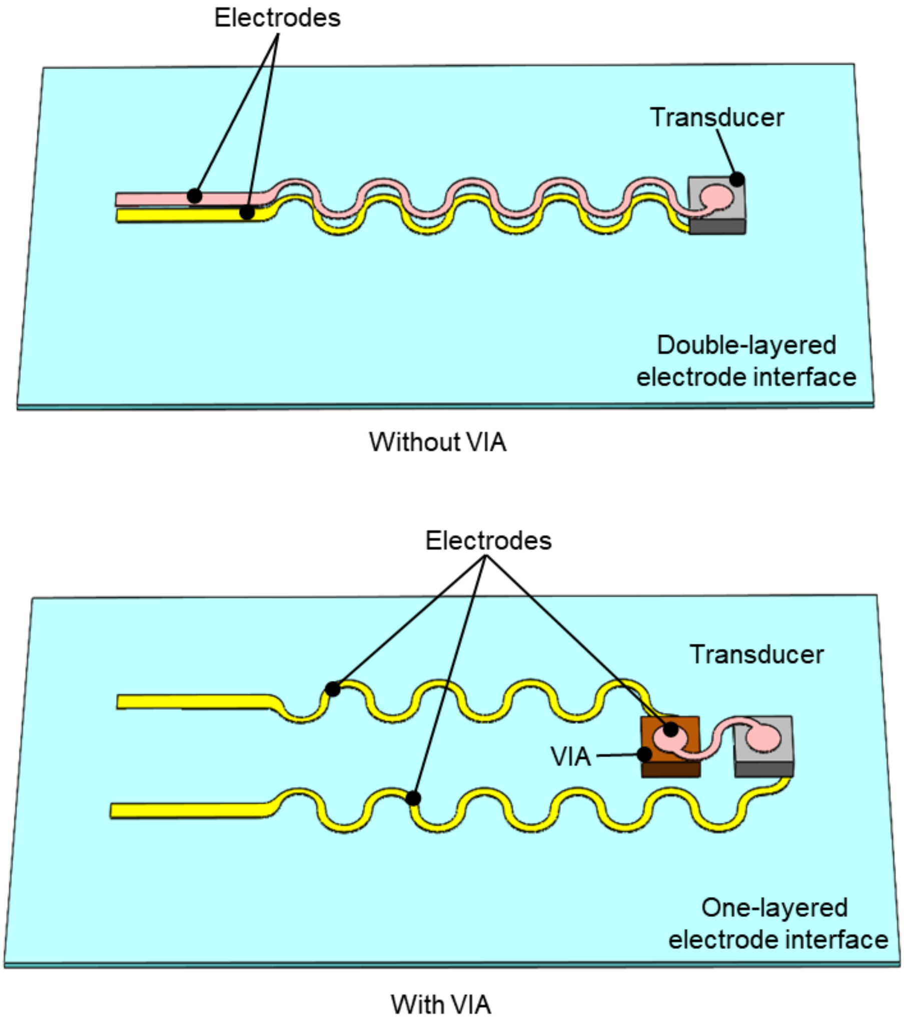

Supplementary Fig. 6. Structure of the ultrasound sensor without and with VIA.

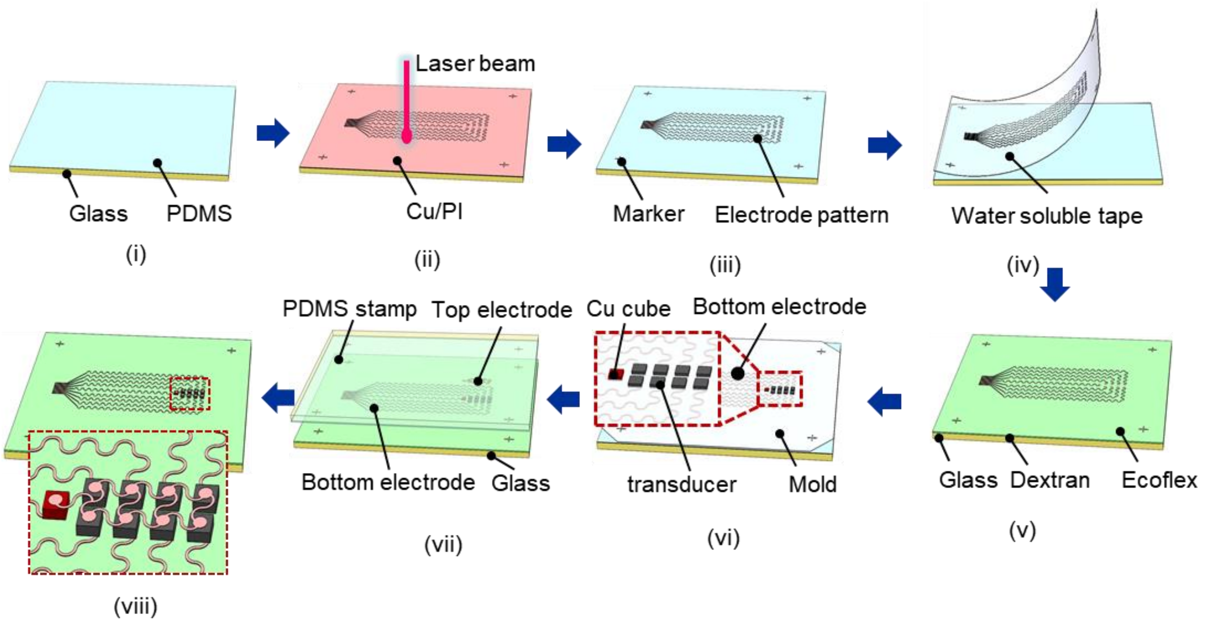

Supplementary Fig. 7. Fabrication process of ultrasound sensor array (DEVICE 1). (i) Spin coat PDMS on glass slide; (ii) laminate Cu/PI foil on PDMS substrate, and define electrode pattern by laser cutter; (iii) remove excess electrode material; (iv) peel off electrode by water soluble tape; (v) transfer bottom electrode on Ecoflex substrate spin-coated on dextran-decorated glass slide; (vi) bond transducer and Cu cube on bottom electrode; (vii) align top electrode with the transducers using PDMS stamp; (viii) bond the top electrode and remove PDMS stamp.

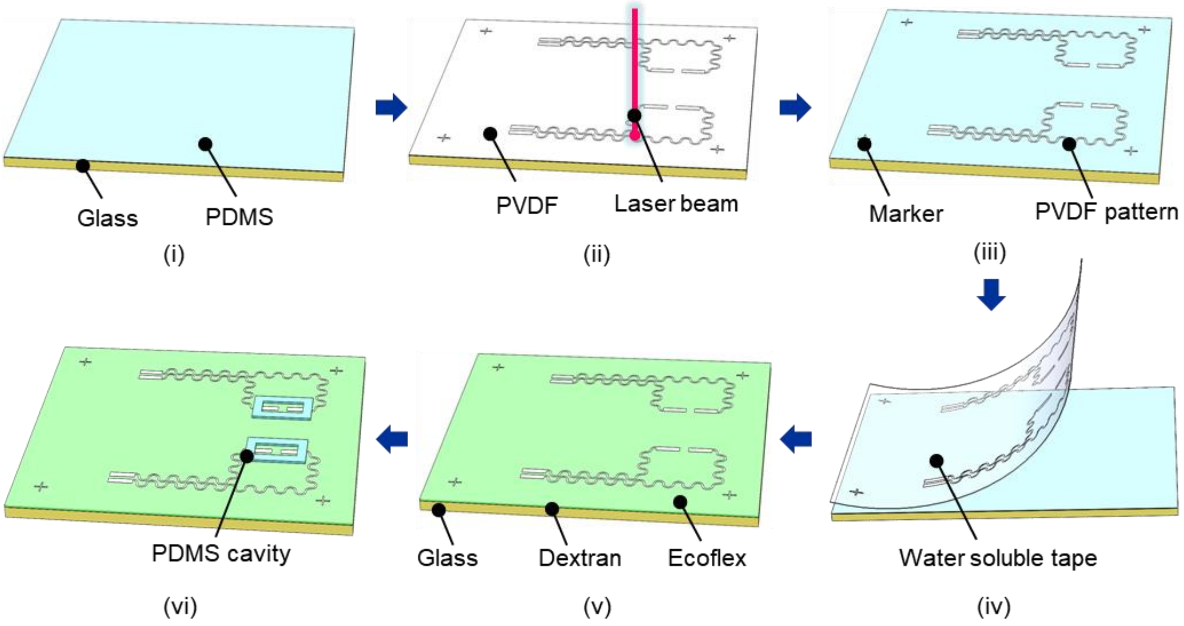

Supplementary Fig. 8. Fabrication process of PWF sensor (DEVICE 2). (i) Spin coat PDMS on glass slide; (ii) laminate PVDF film on PDMS, and define pattern by laser cutter; (iii) remove excess PVDF material; (iv) peel off PWF sensor by water soluble tape; (v) transfer PWF sensor on Ecoflex substrate spin-coated on dextran-decorated glass slide; (vi) bond PDMS cavity on PWF sensor.

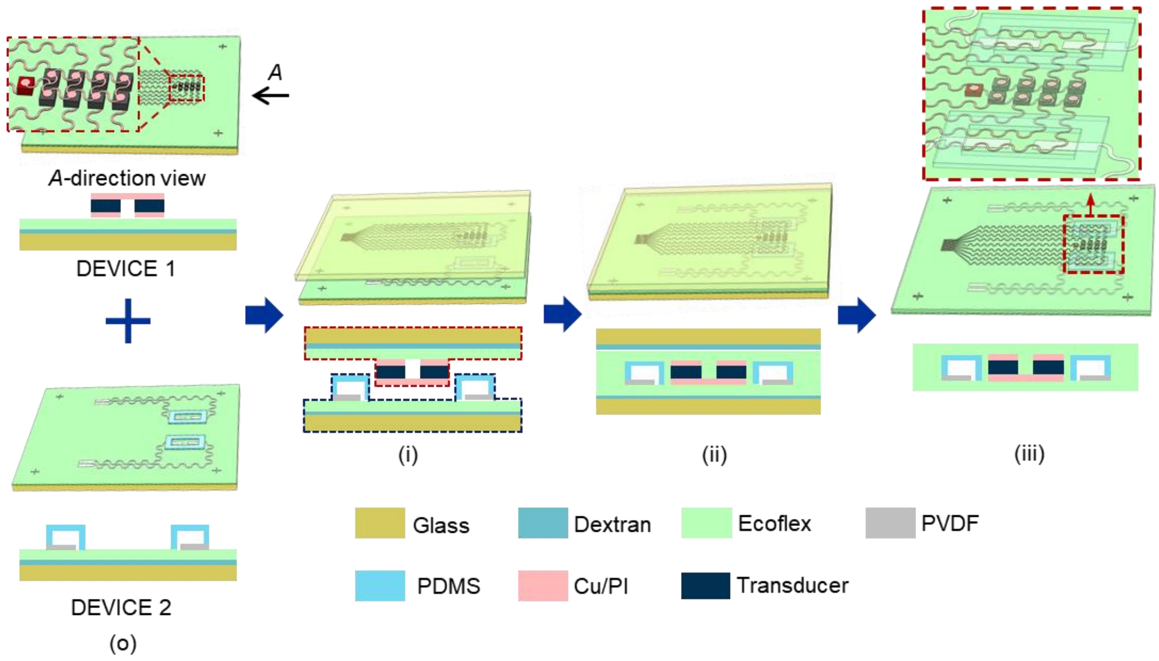

Supplementary Fig. 9. Fabrication process of dual-model CSPM. (i) Align ultrasound sensor array with the PWF sensor; (ii) pour Ecoflex to fulfill the gaps between two glass slides; (iii) remove the glass slides after the Ecoflex cures.

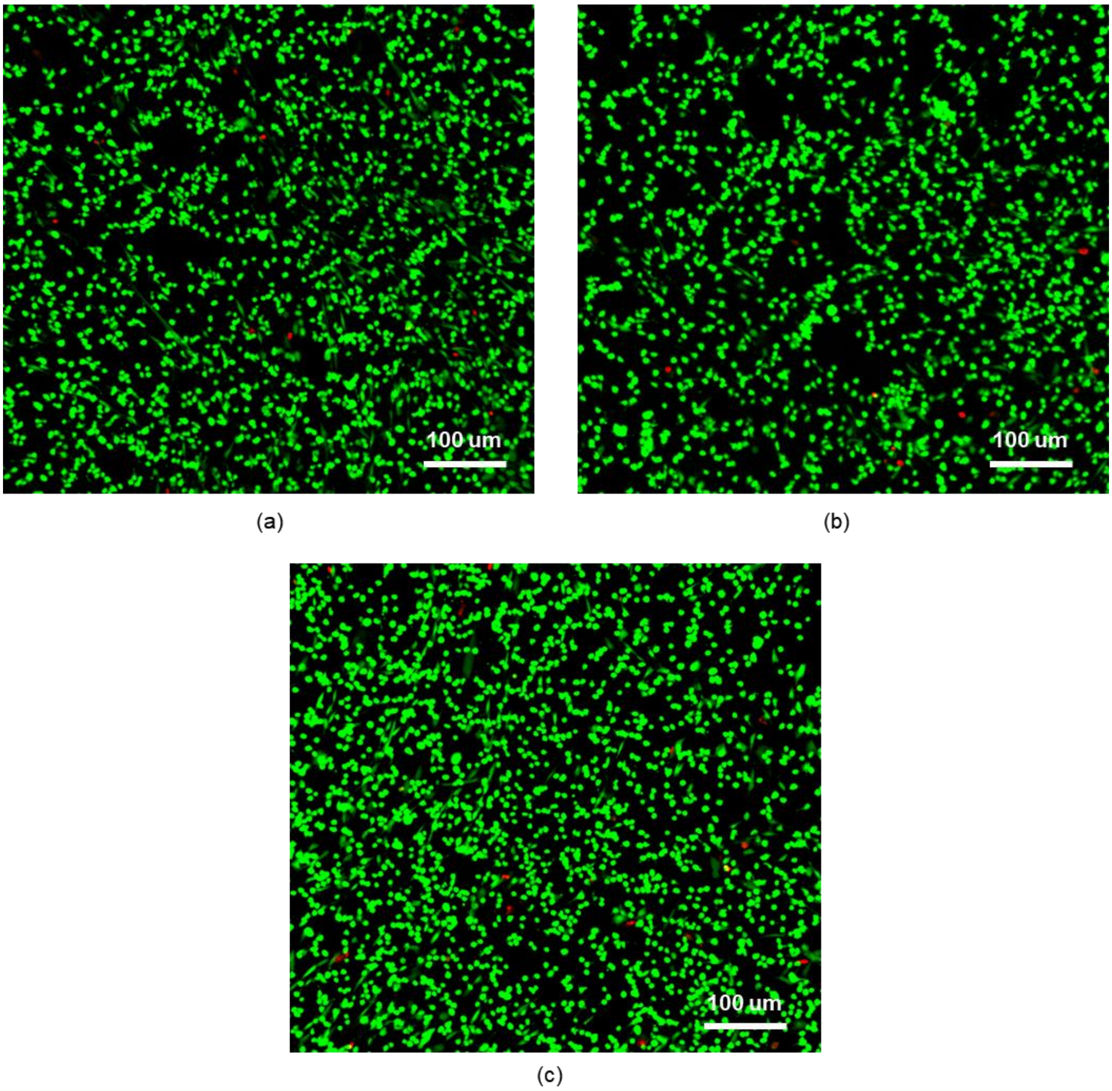

Supplementary Fig. 10. Fluorescent images of the cells after 0h (a), 12h (b) and 24 h (c) continuous exposure to the device.

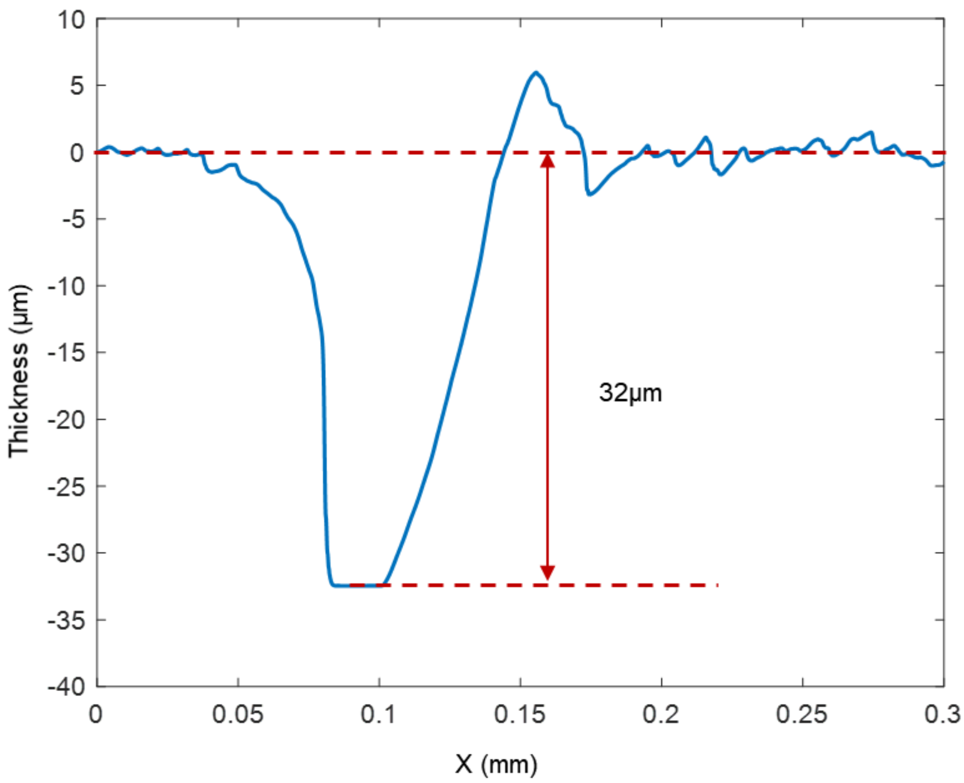

Supplementary Fig. 11. Thickness of Ecoflex 00-30 at speed of 1500 rpm.

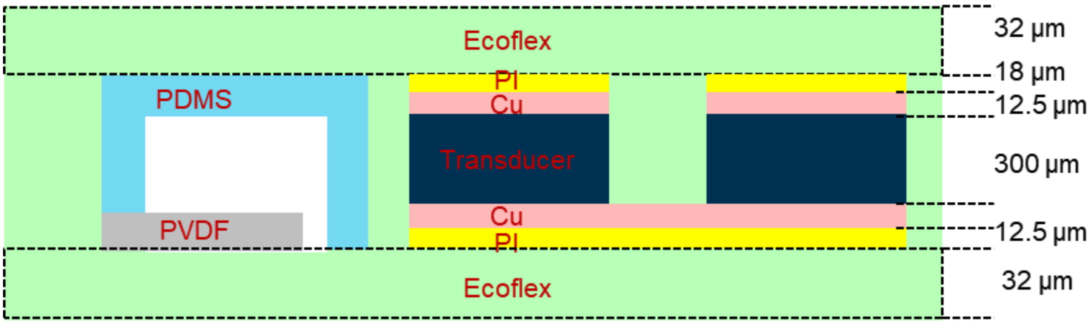

Supplementary Fig. 12. Cross-sectional view of the device with thickness dimensions.

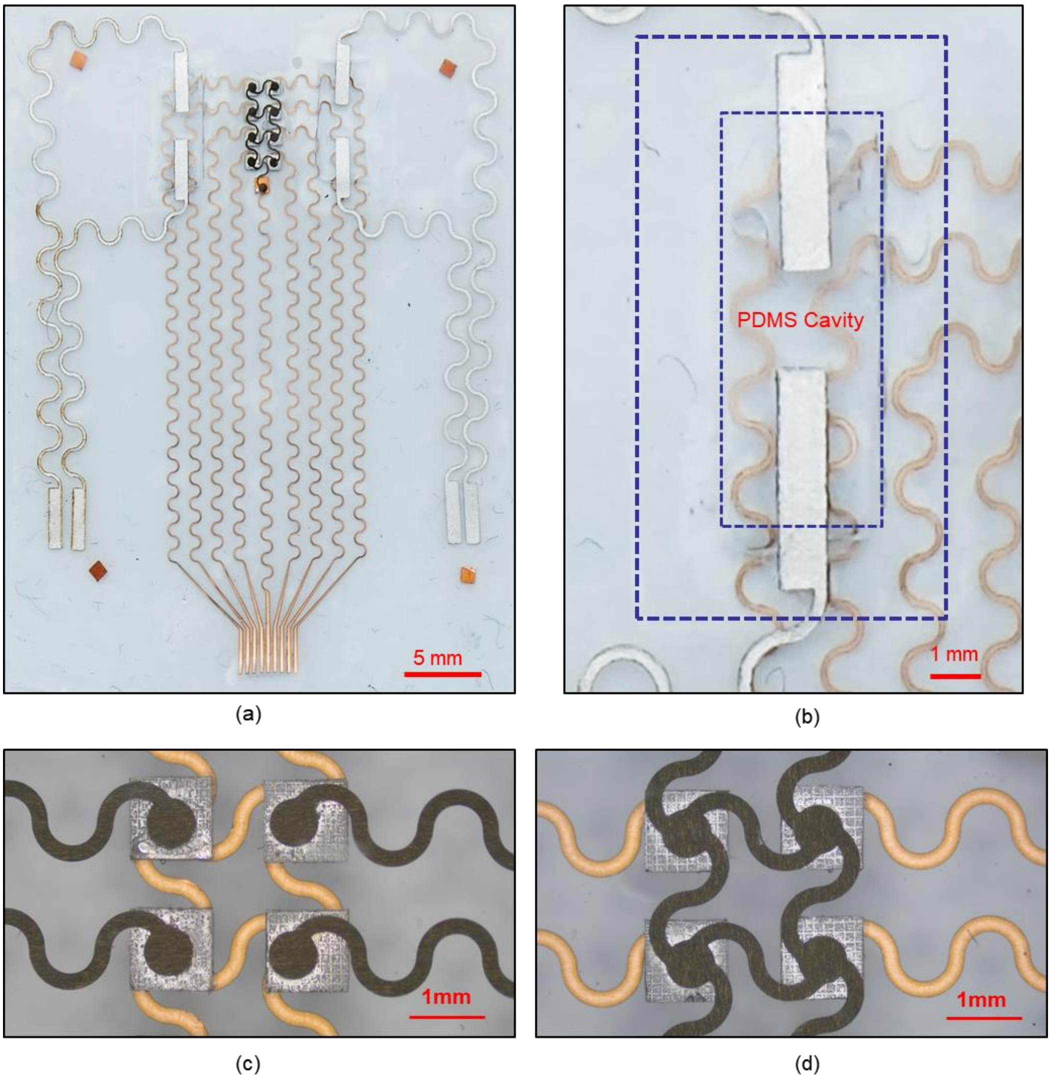

Supplementary Fig. 13. Optical images of the CSPM. (a) Optical image showing the overall device. (b) Optical image showing the PWF sensor with PDMS cavity. (c) Top view of ultrasound sensor. (d) Back view of ultrasound sensor.

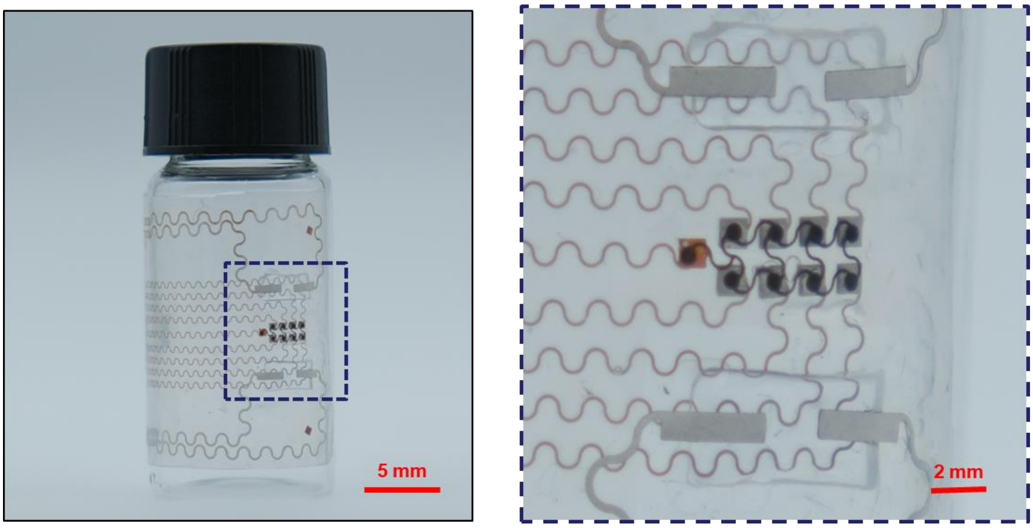

Supplementary Fig. 14. Optical images of the CSPM attached on a sample bottle with radius of ~1.36 mm.

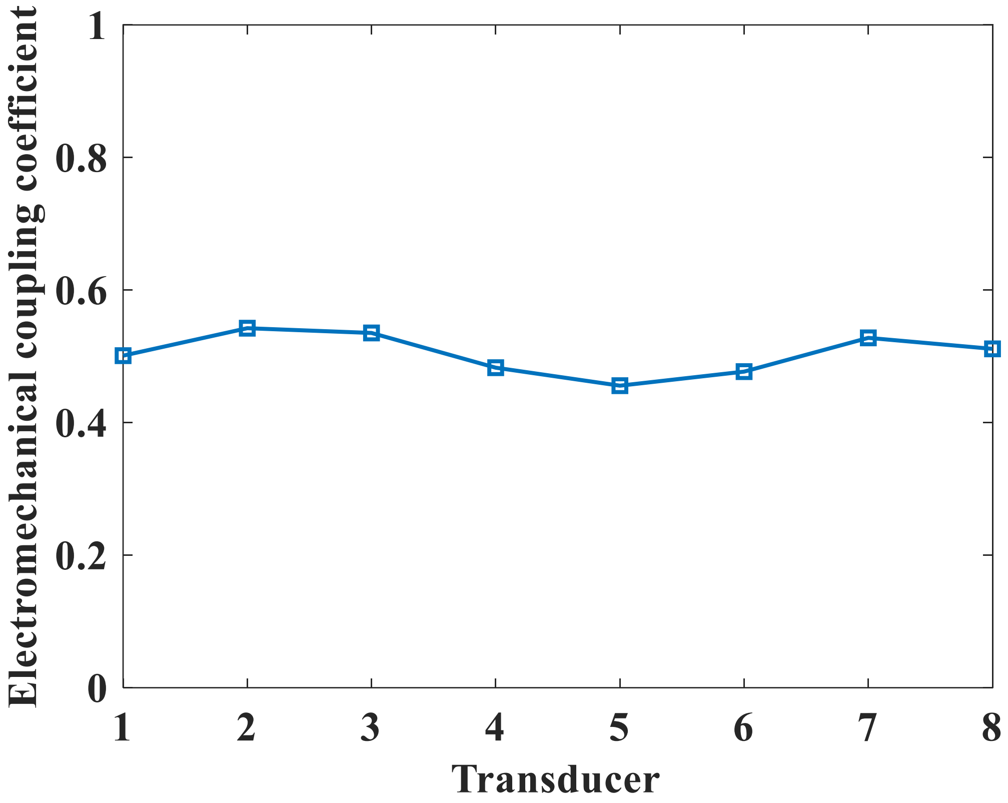

Supplementary Fig. 15. Electromechanical coupling coefficient of eight transducers.

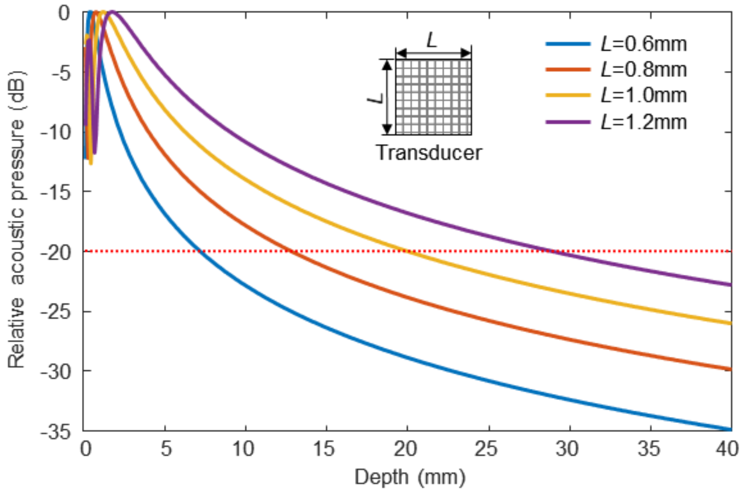

Supplementary Fig. 16. Relative acoustic pressure vs depth along the center line of the ultrasound transducers.

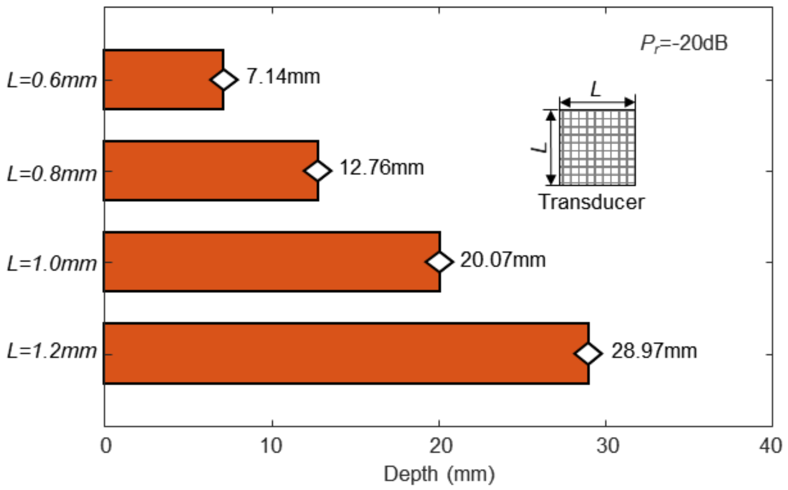

Supplementary Fig. 17. The depth of the ultrasound wave as relative acoustic pressure is -20dB.

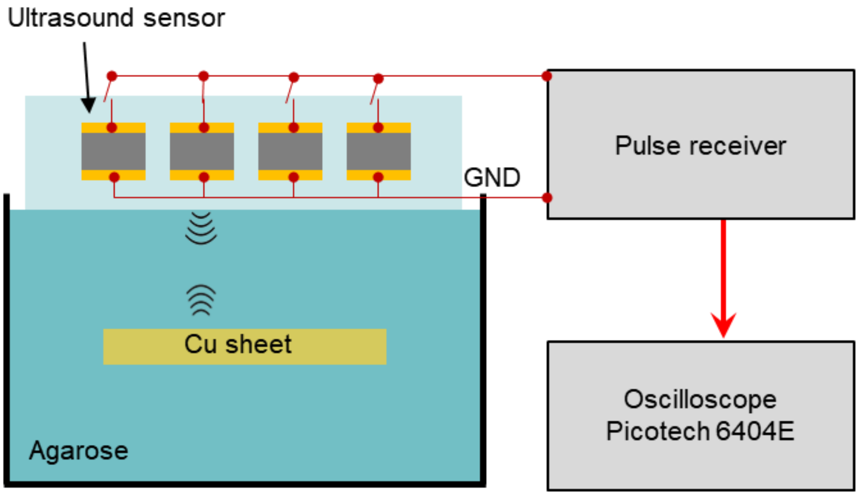

Supplementary Fig. 18. Echo test setup of ultrasound transducer.

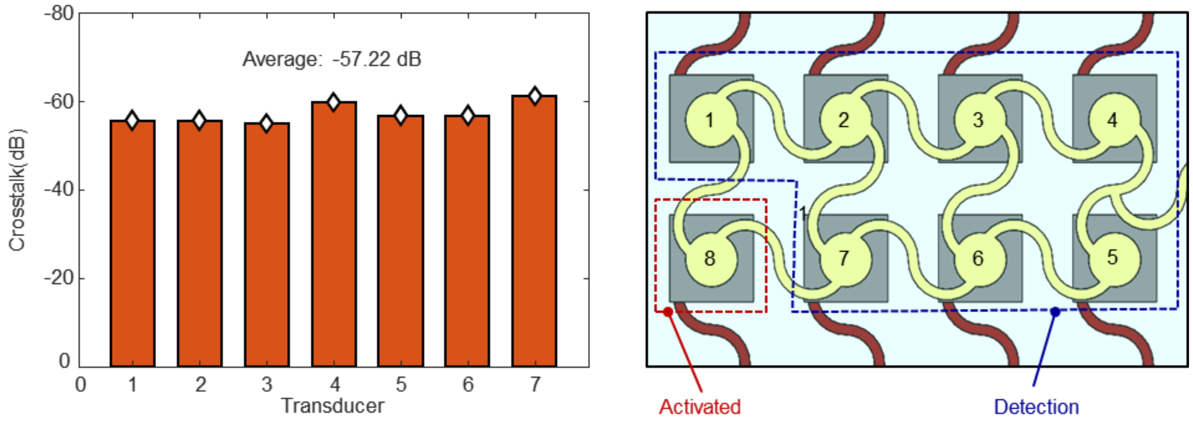

(a) (b)

Supplementary Fig. 19. Cross-talk test of ultrasound transducer array. (a) Cross-talk levels between transducers. (b) cross-talk test diagram.

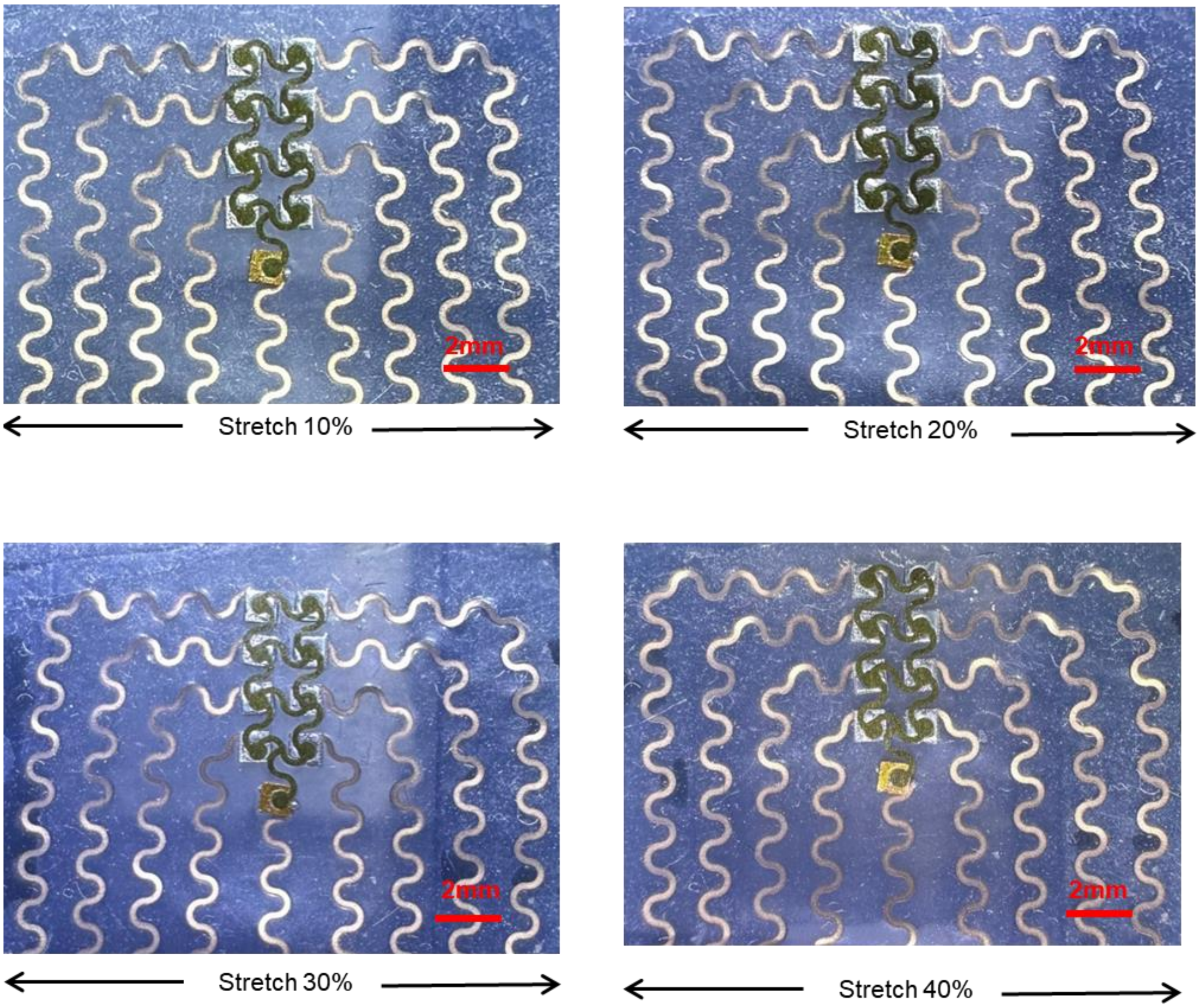

Supplementary Fig. 20. The optical image of ultrasound sensor array under 10%, 20%, 30% and 40% tensile strain.

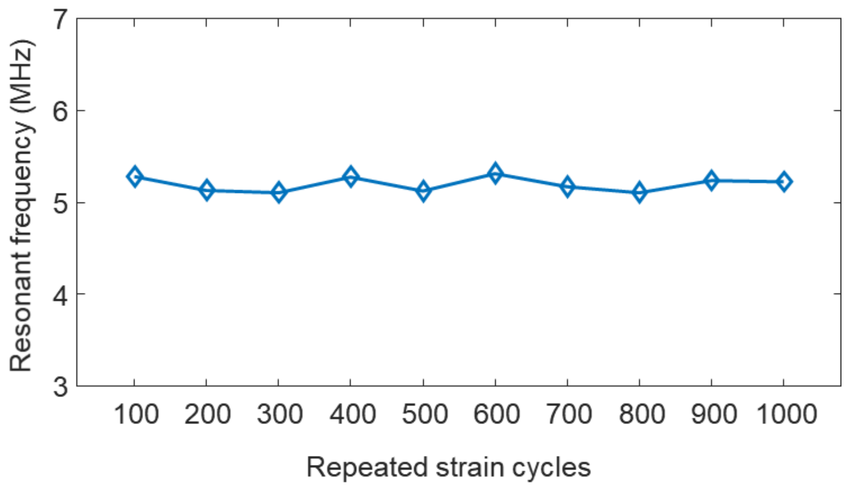

Supplementary Fig. 21. Resonant frequency of ultrasound sensor under repeated strain cycles.

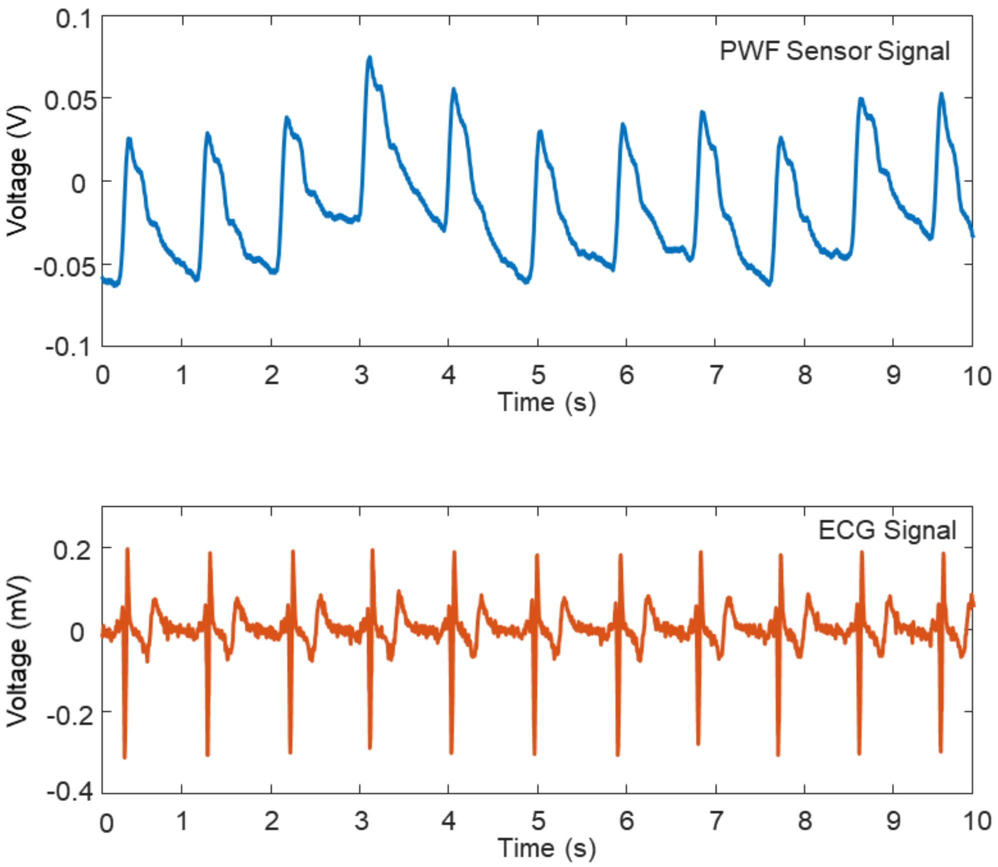

Supplementary Fig. 22. Results of the synchronization test between the PWF sensor signal and the electrocardiogram signal.

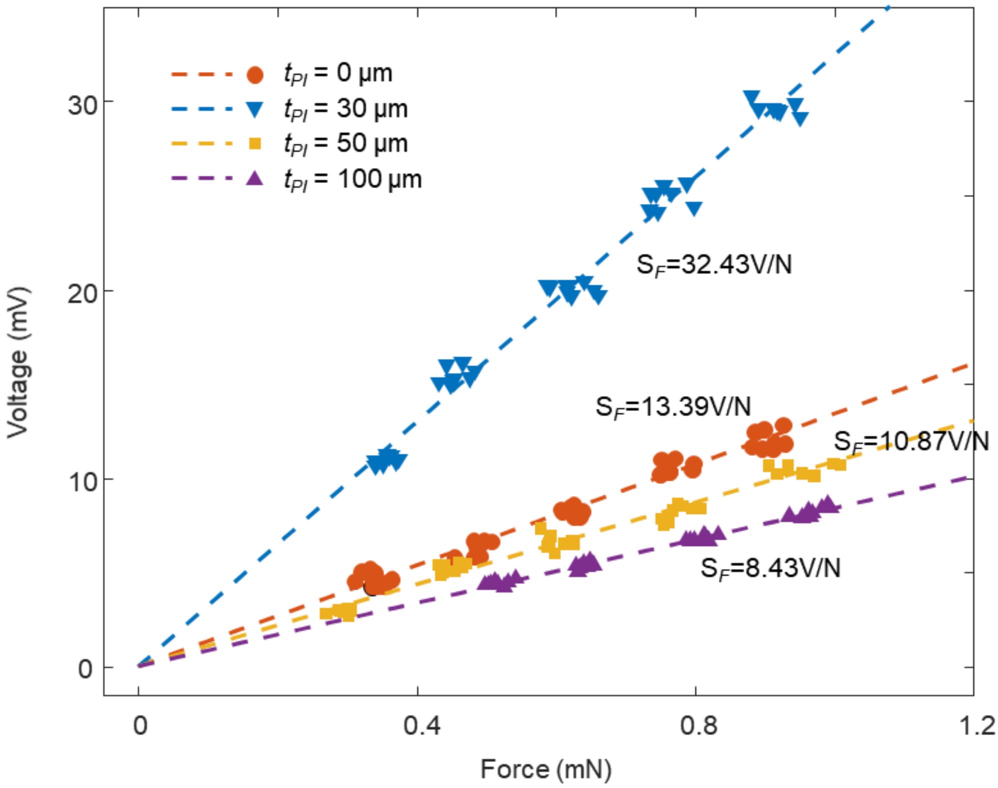

Supplementary Fig. 23. Comparison of PWF sensor with different supporting layer thickness in terms of sensitivity.

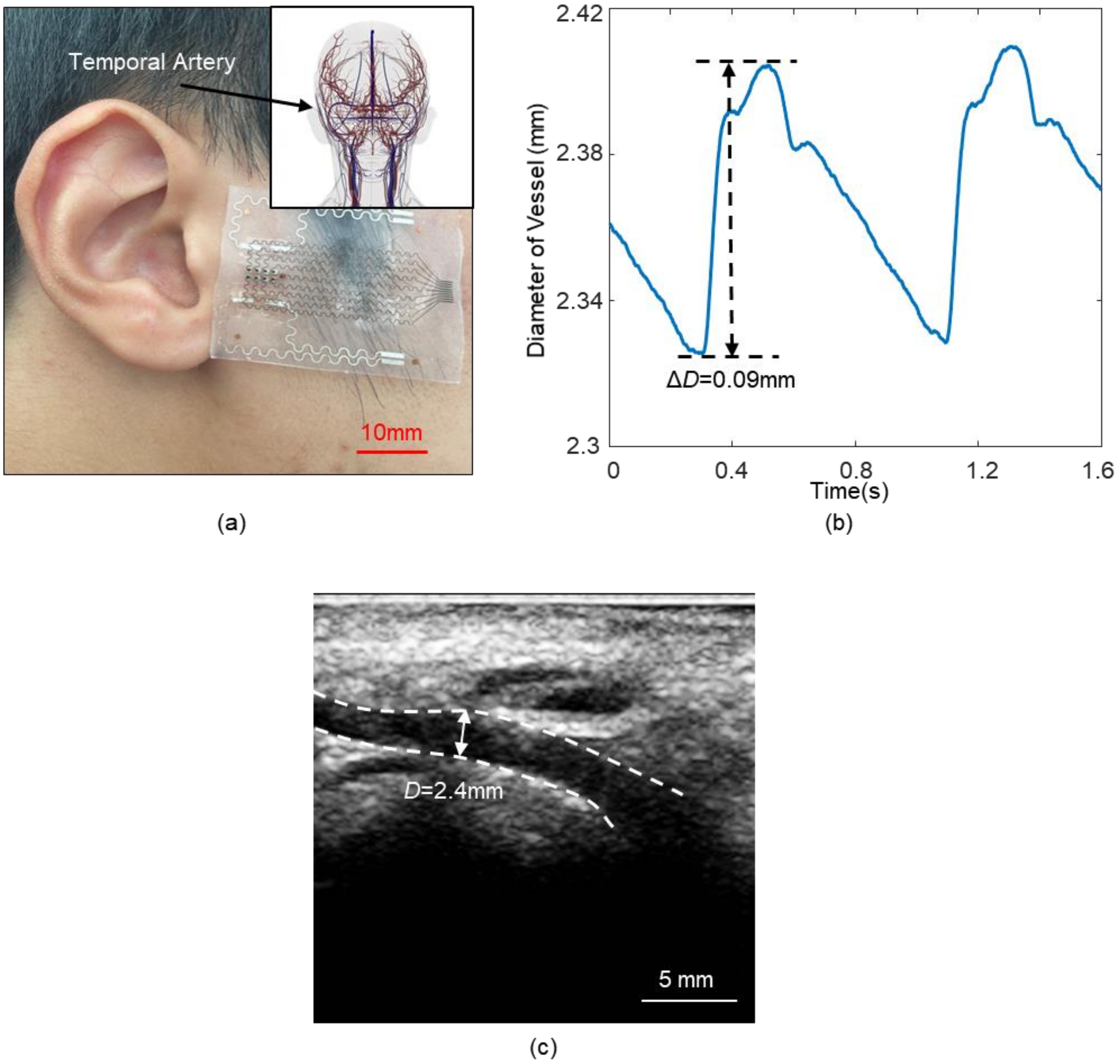

Supplementary Fig. 24. Blood vessel diameter measurement results of our device at temporal artery. a. Optical image of our device on the human temple. Inset shows the vascular structure of the testing site, adapted from https://simvascular.github.io/ (Copyright © SimVascular Development Team, 2023). b. Pulse waveforms of temporal artery, c. Grayscale ultrasound images of temporal artery.

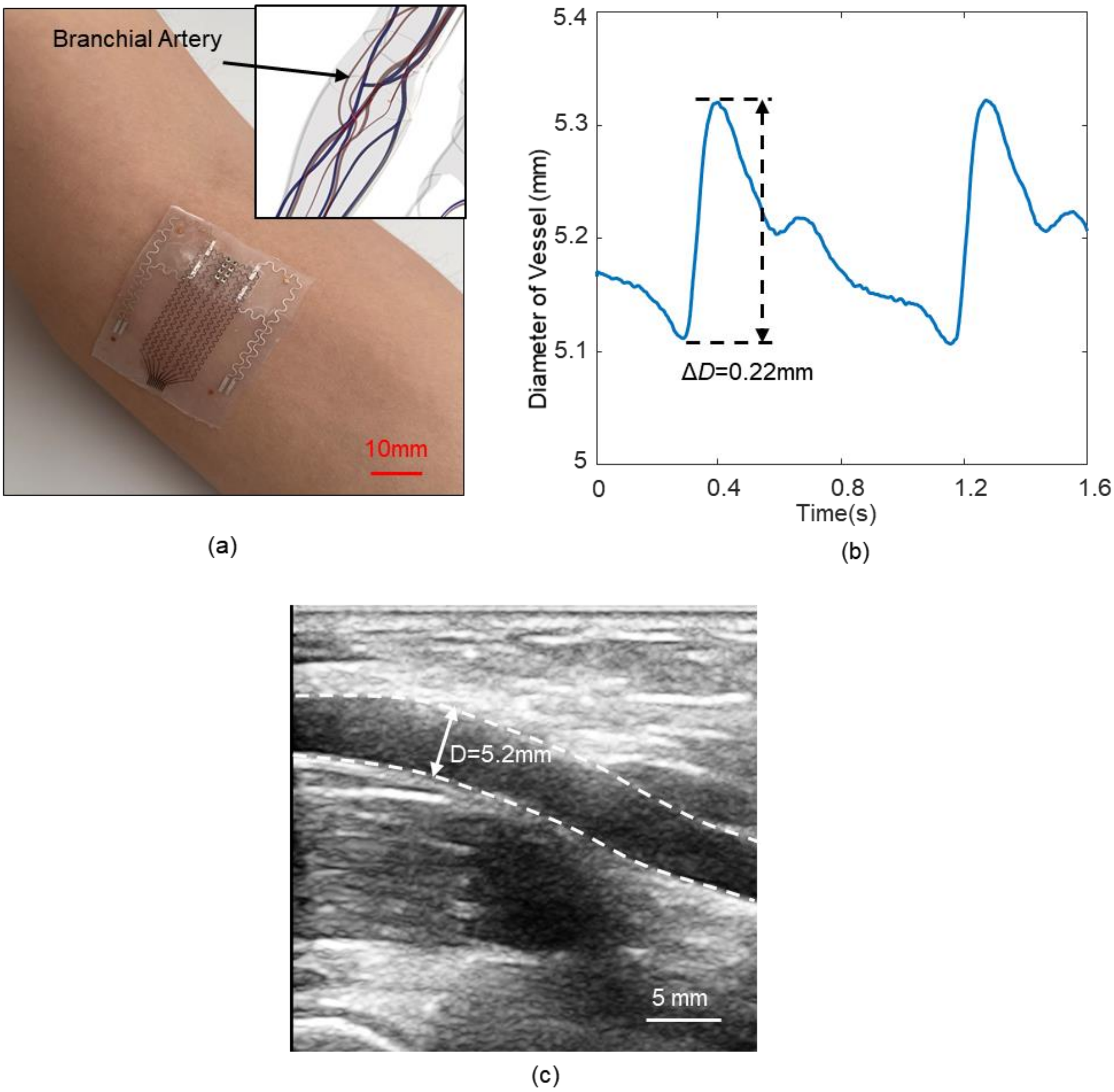

Supplementary Fig. 25. Blood vessel diameter measurement results of our device at brachial artery. a. Optical image of our device on the human arm. Inset shows the vascular structure of the testing site, adapted from https://simvascular.github.io/ (Copyright © SimVascular Development Team, 2023). b. Pulse waveforms of t brachial artery, c. Grayscale ultrasound images of brachial artery.

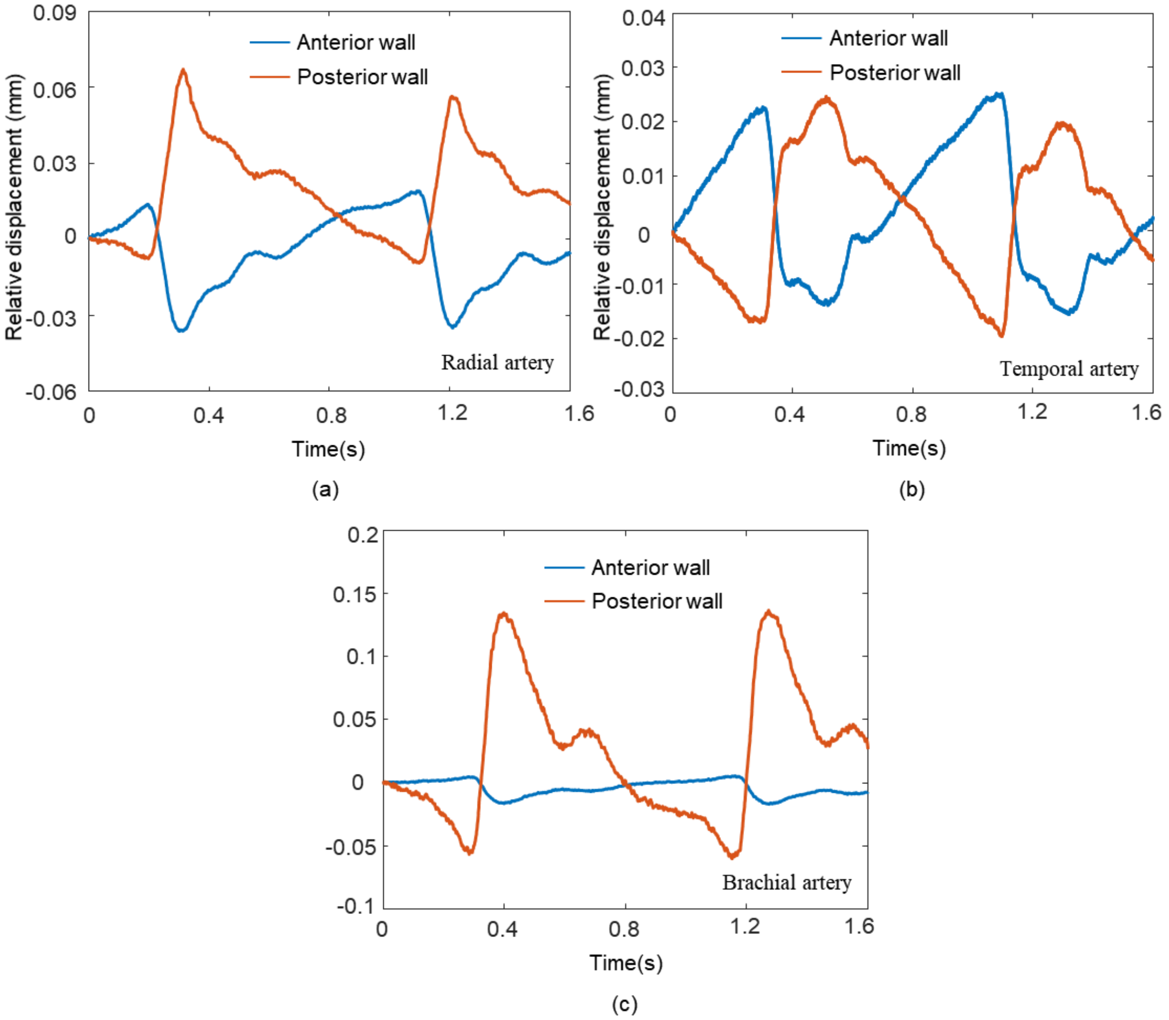

Supplementary Fig. 26. Movement of artery's posterior wall and anterior wall at different anatomical sites, including a. radial artery, b. temporal artery and c. brachial artery.

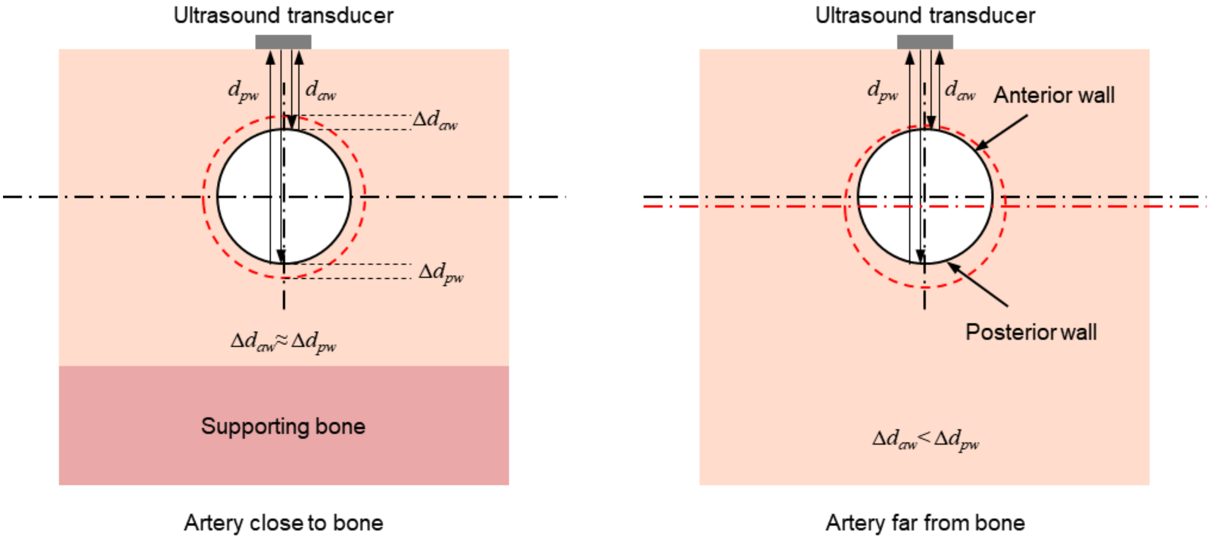

Supplementary Fig. 27. Diagram illustrating the movement of artery's posterior wall and anterior wall.

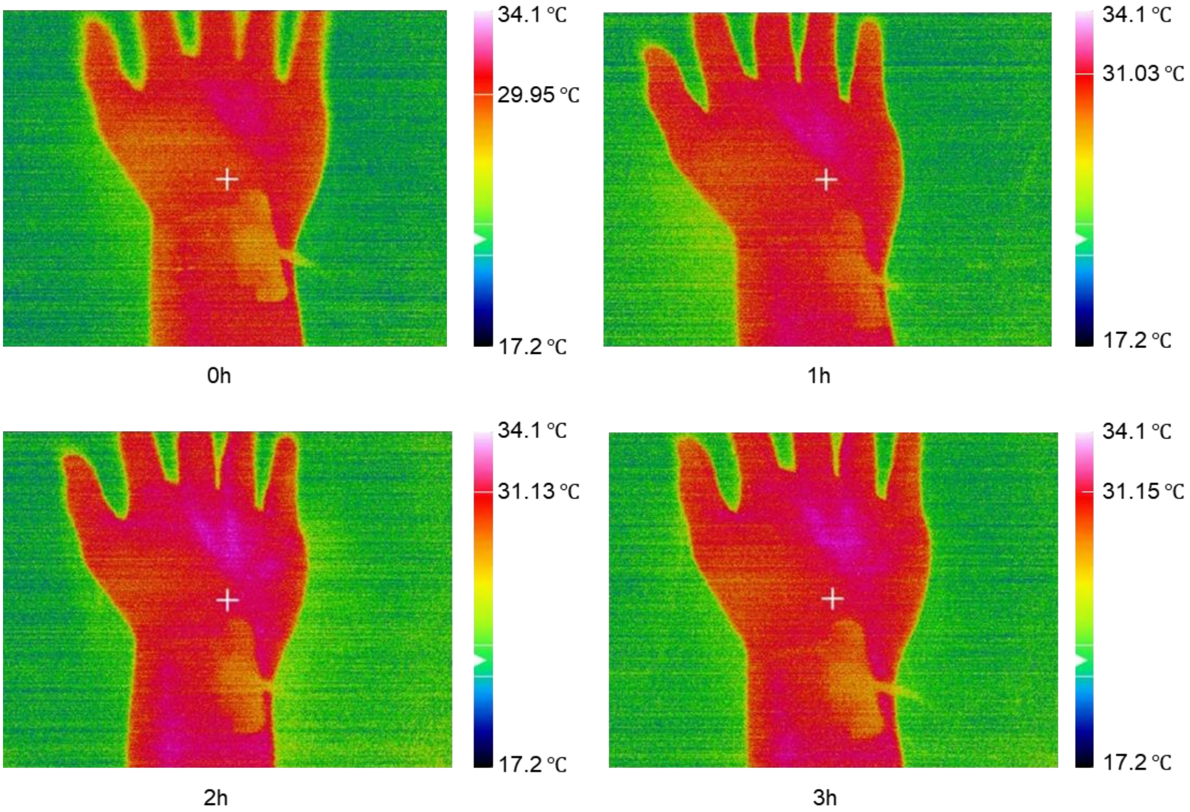

Supplementary Fig. 28. The thermal distribution of the device at the outset, after 1 hour, 2 hours, and 3 hours of operation.

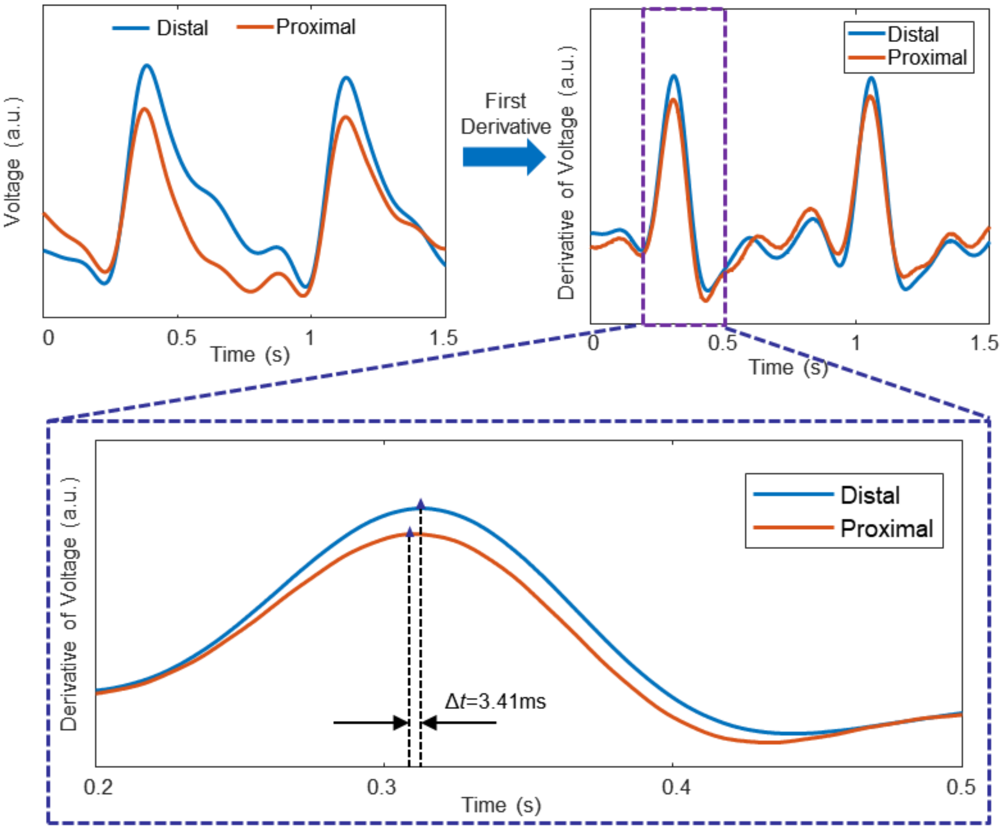

Supplementary Fig. 29. Measured two pulse waveforms, the first derivative of the measured two pulse waveforms and its zoomed-in view, showing the time difference between the peaks of the first derivative results is 3.41ms.

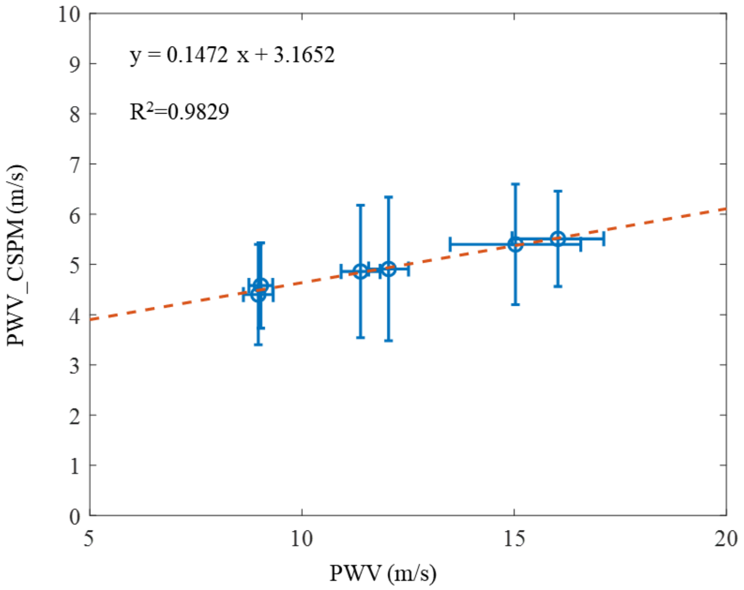

Supplementary Fig. 30. Relationship between PWV and PWV_CSPM.

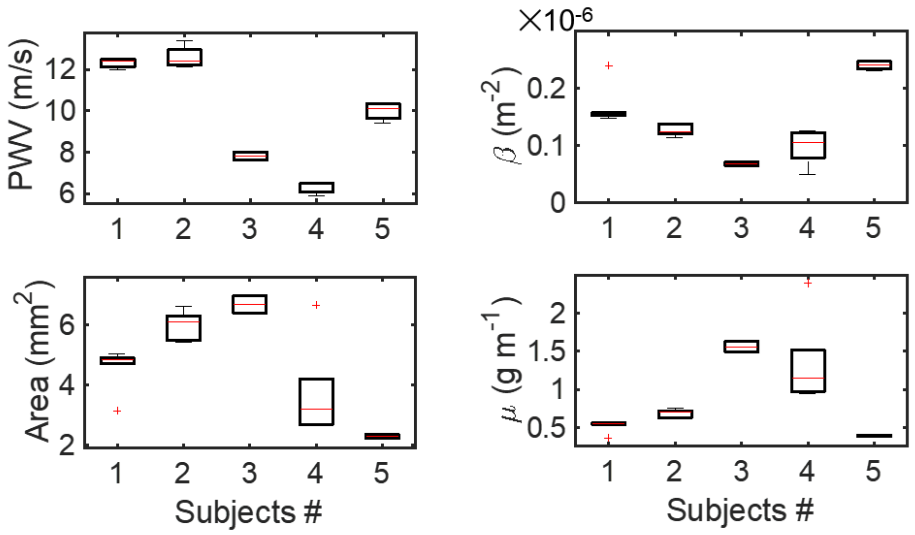

Supplementary Fig. 31. Personalized PWV, β stiffness, blood vessel cross-sectional area, and linear blood density μ of five subjects.

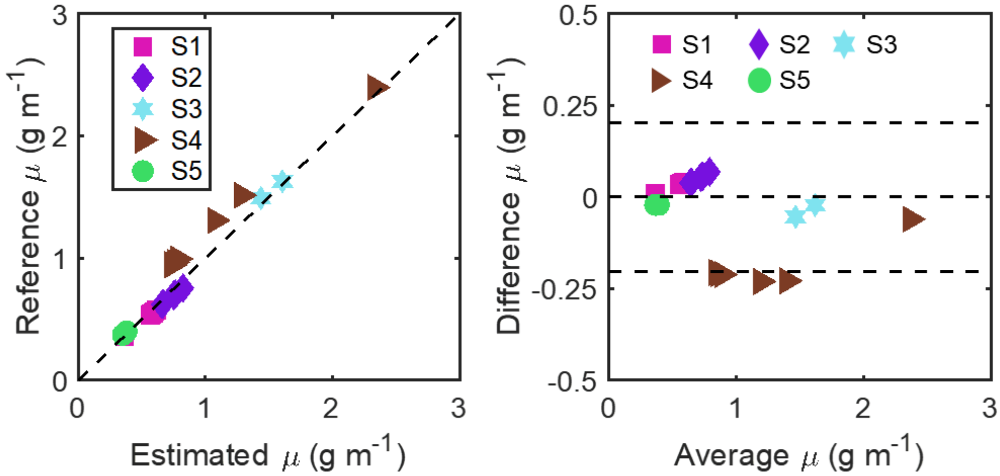

Supplementary Fig. 32. Relationship between the estimated μ and reference μ (left) showing good consistency (p<0.0001), and Bland-Altman plots with bias (black dashed line) and ±1.96 s.d. (dashed grey lines, confidence intervals (CI)) for measurements of linear blood density μ (right).

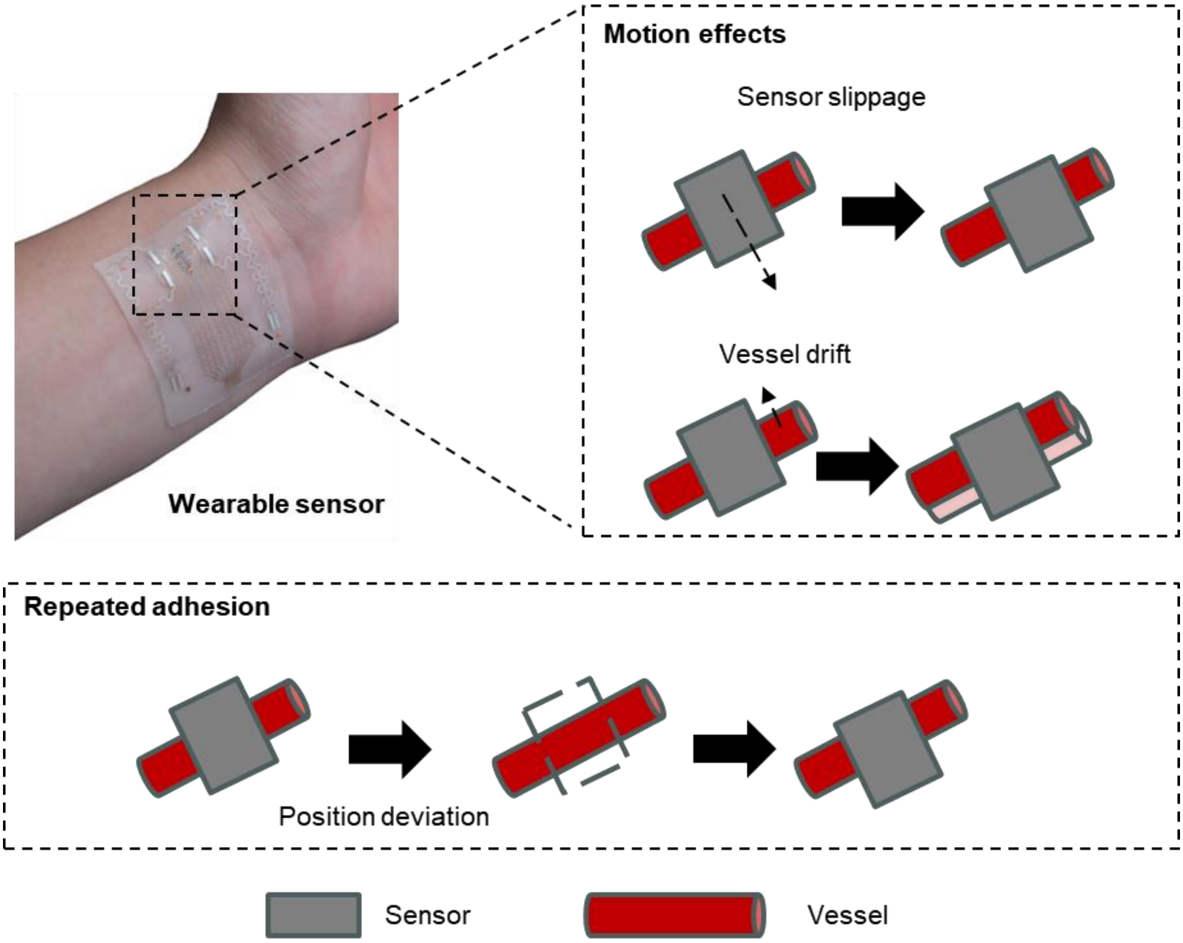

Supplementary Fig. 33. The relative position offset between wearable device and vessel.

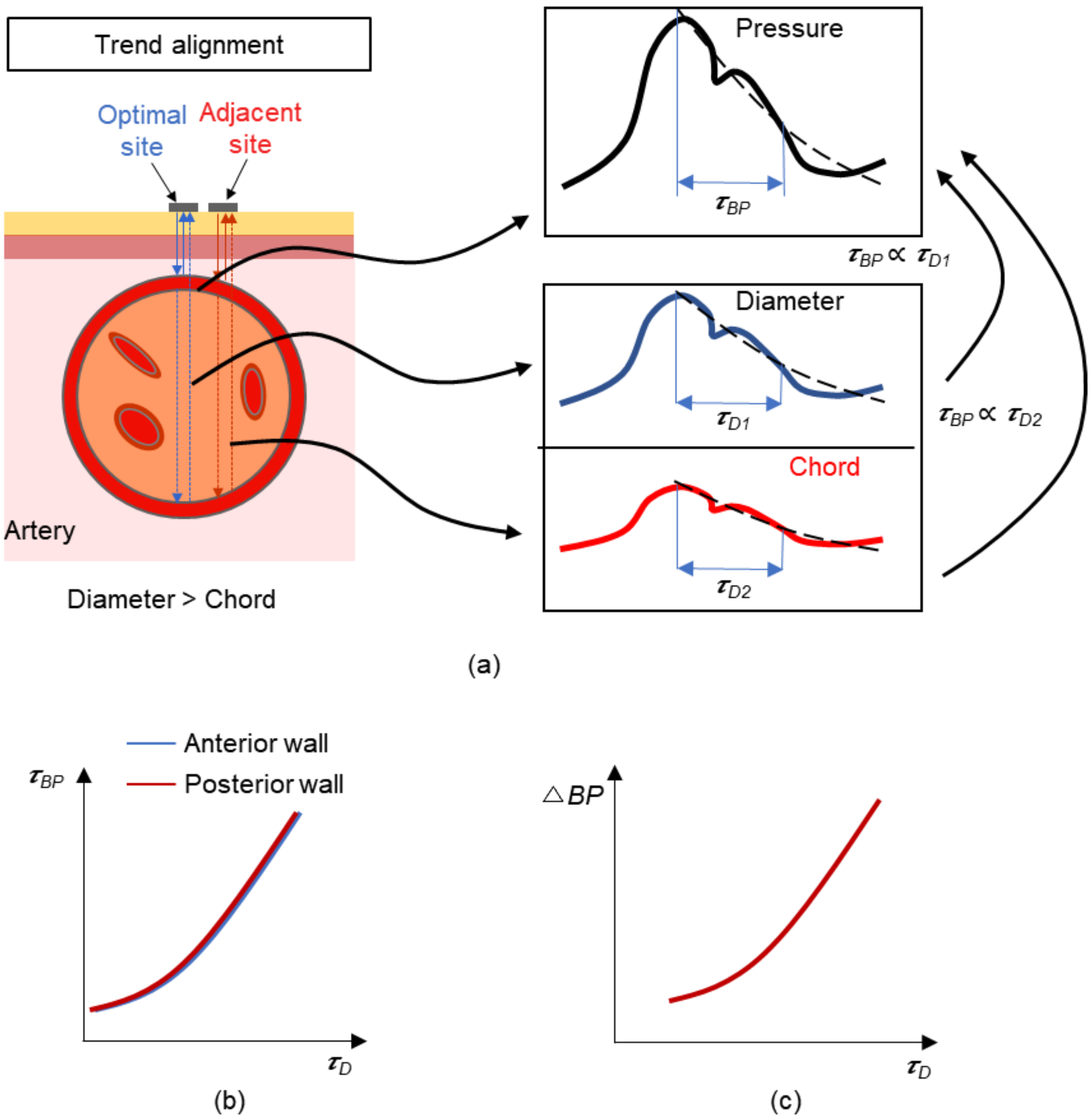

Supplementary Fig. 34. The principle of the time-decay method for continuous bp trend alignment. a. The time constant of BP is proportional to the time constant of the diameter of the artery. Similarly, the time constant of BP is also proportional to the time constant of the chord of the artery. Therefore, the dynamic changes in BP can be traced by monitoring the changes in the time constant of the artery, even if the sensor is attached at an adjacent site. b. The relationship between the time constant of the diameter and BP. c. The relationship between the time constant of the diameter and changes in BP values.

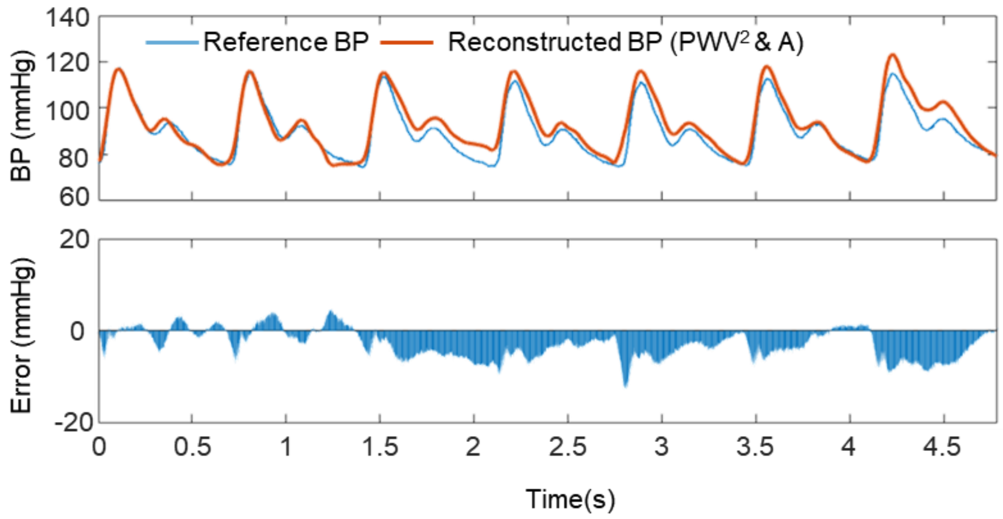

Supplementary Fig. 35. Results of continuous BP tracking by PWV^2&A method.

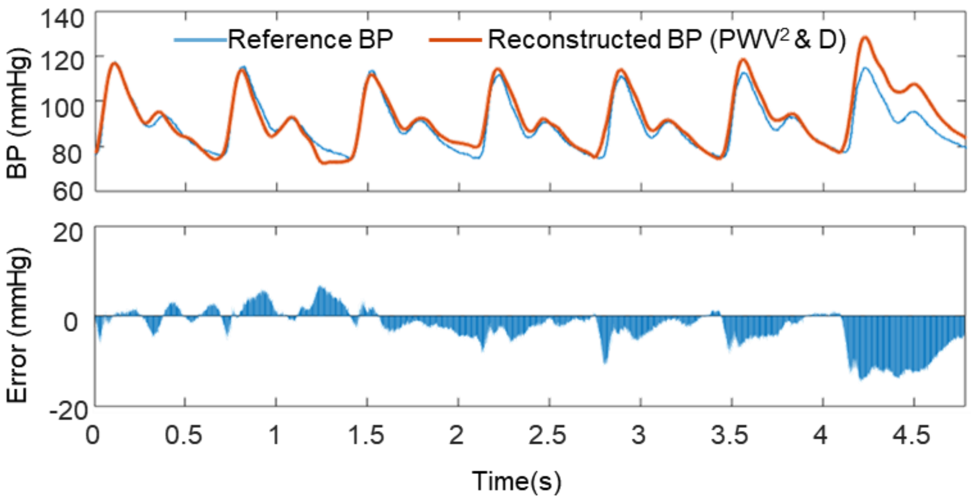

Supplementary Fig. 36. Results of continuous BP tracking by PWV^2& D method.

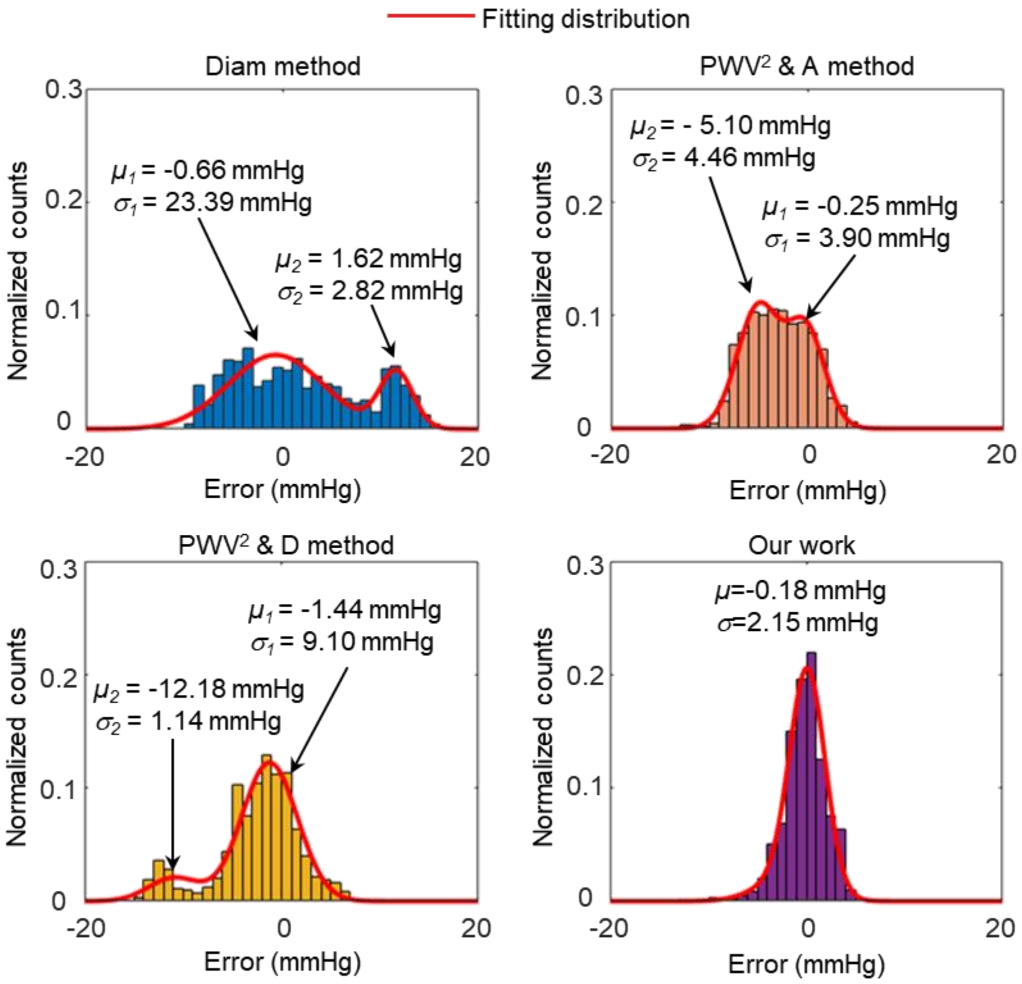

Supplementary Fig. 37. Error statistics results of BP tracking by different continues BP algorithms.

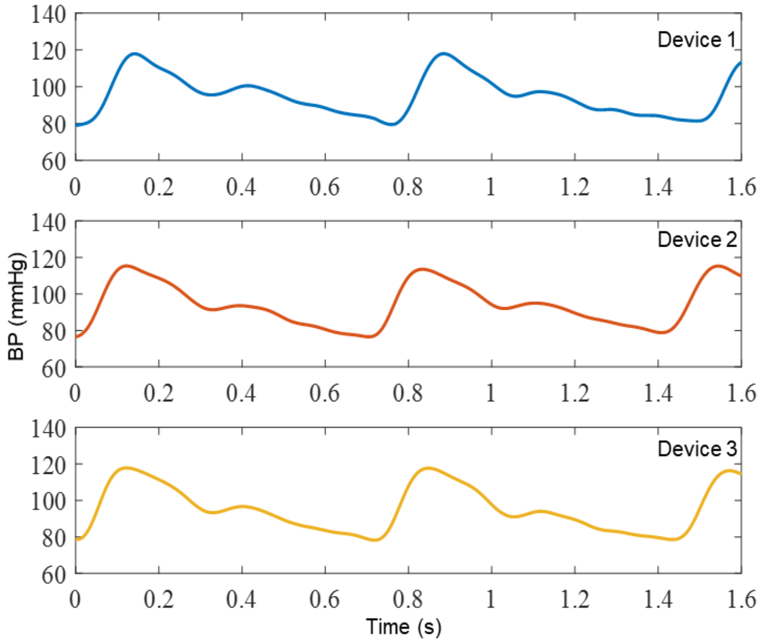

Supplementary Fig. 38. BP results of three different CSPMs.

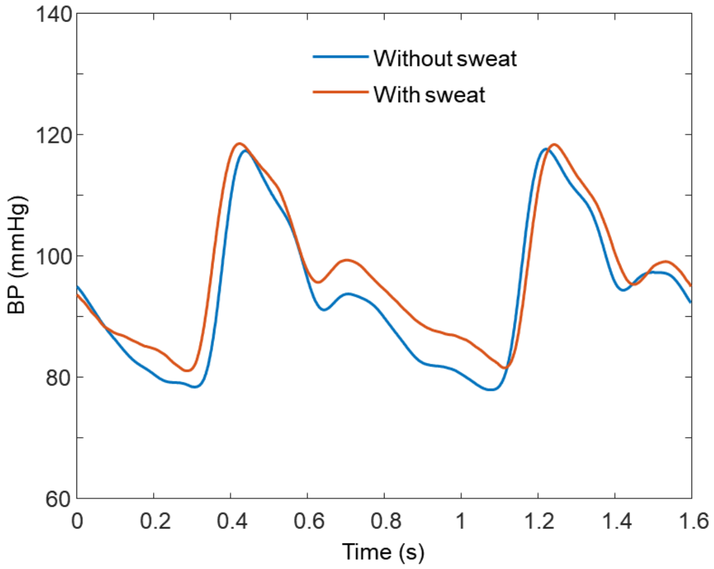

Supplementary Fig. 39. BP results of CSPM with and without sweat.

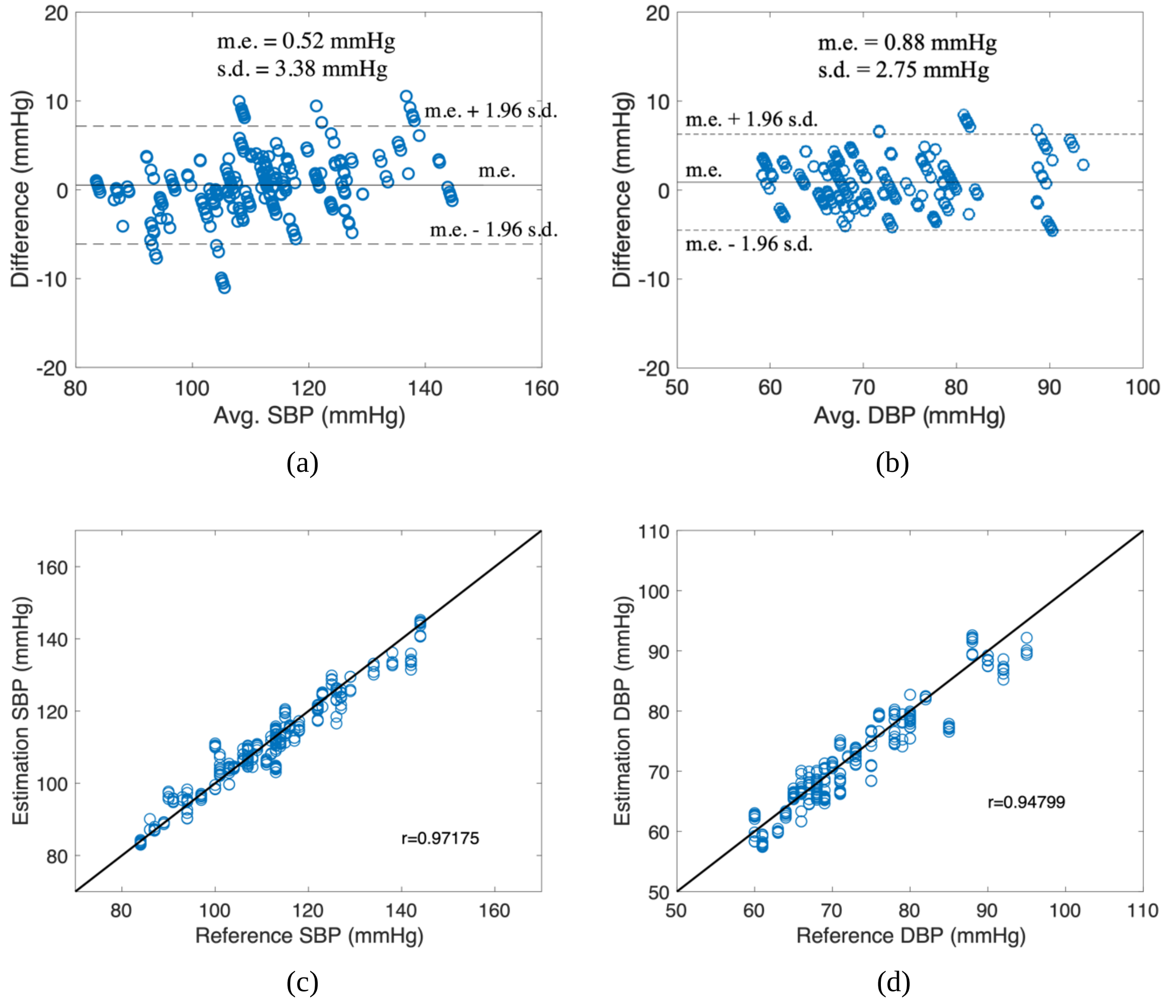

Supplementary Fig. 40. Bland-Altman plots to validate the accuracy for SBP (a) and DBP (b) across diverse subpopulations with individual calibration. Correlation plots of the reference SBP (c) and DBP (d) and the corresponding BP estimations by our BP model across diverse subpopulations with individual calibration.

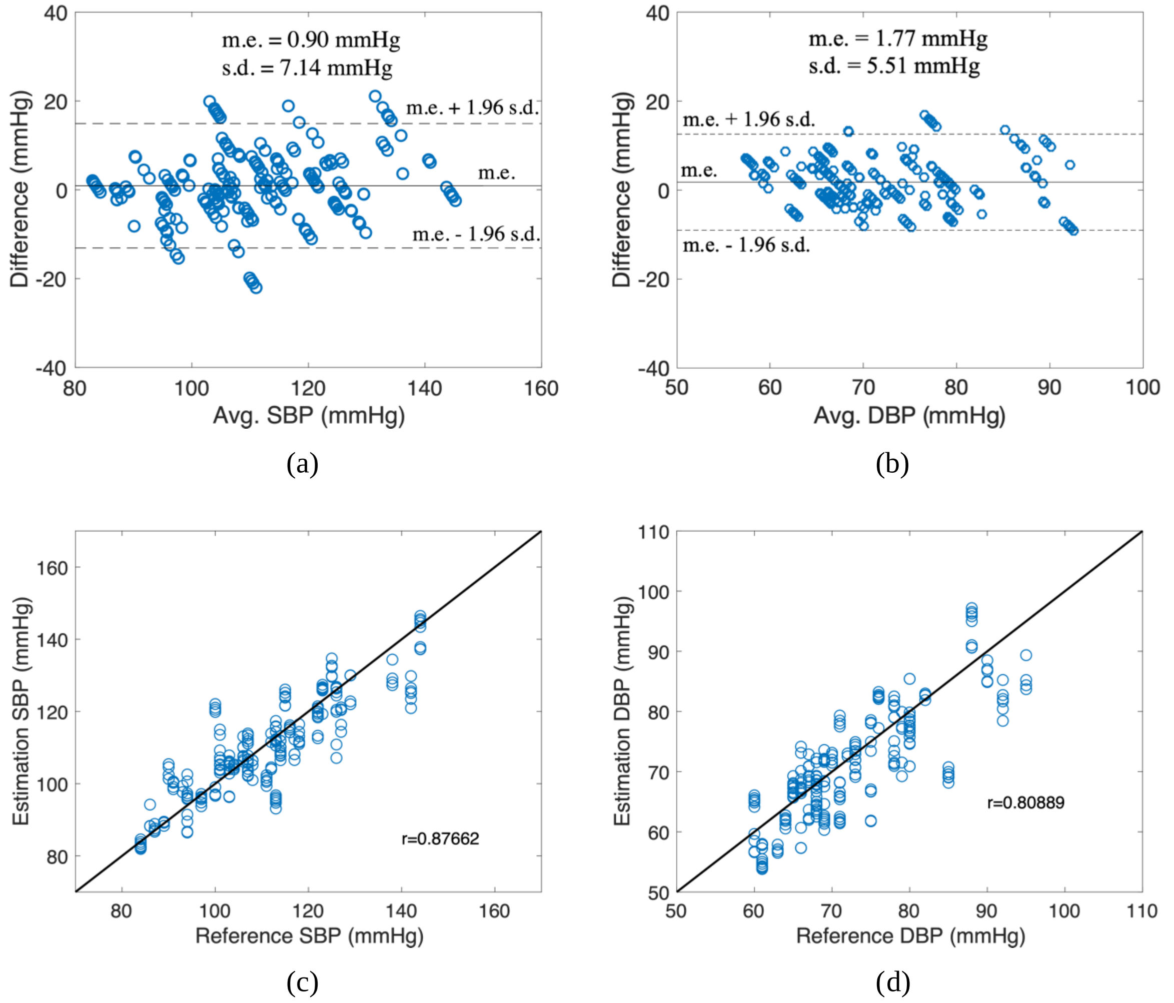

Supplementary Fig. 41. Bland-Altman plots to validate the accuracy for SBP (a) and DBP (b) across diverse subpopulations without individual calibration. Correlation plots of the reference SBP (c) and DBP (d) and the corresponding BP estimations by our BP model across diverse subpopulations with individual calibration.

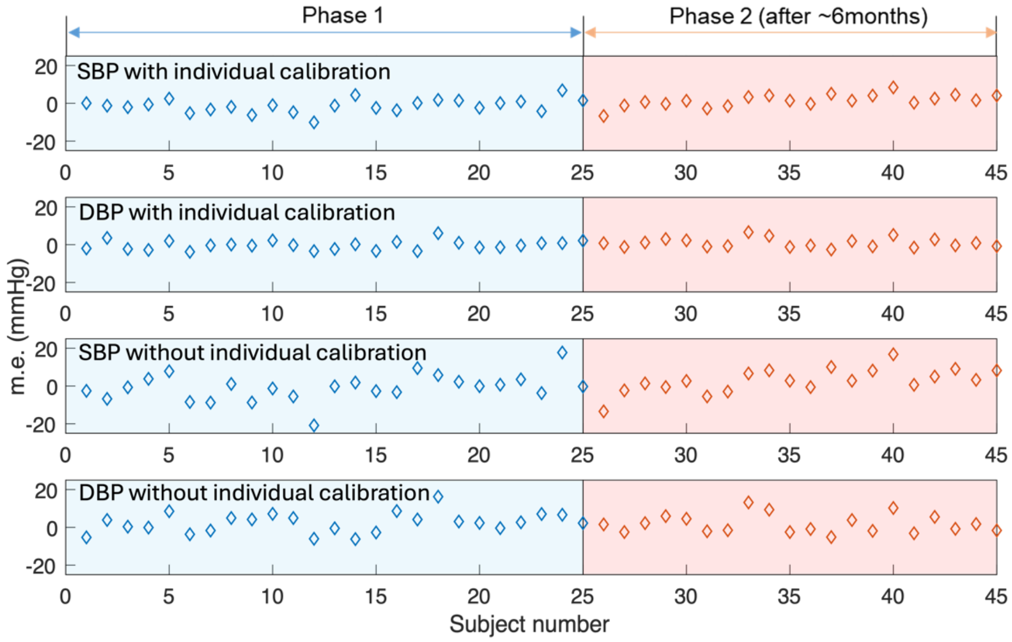

330

331 **Supplementary Fig. 42. Mean error of BP across diverse populations.**

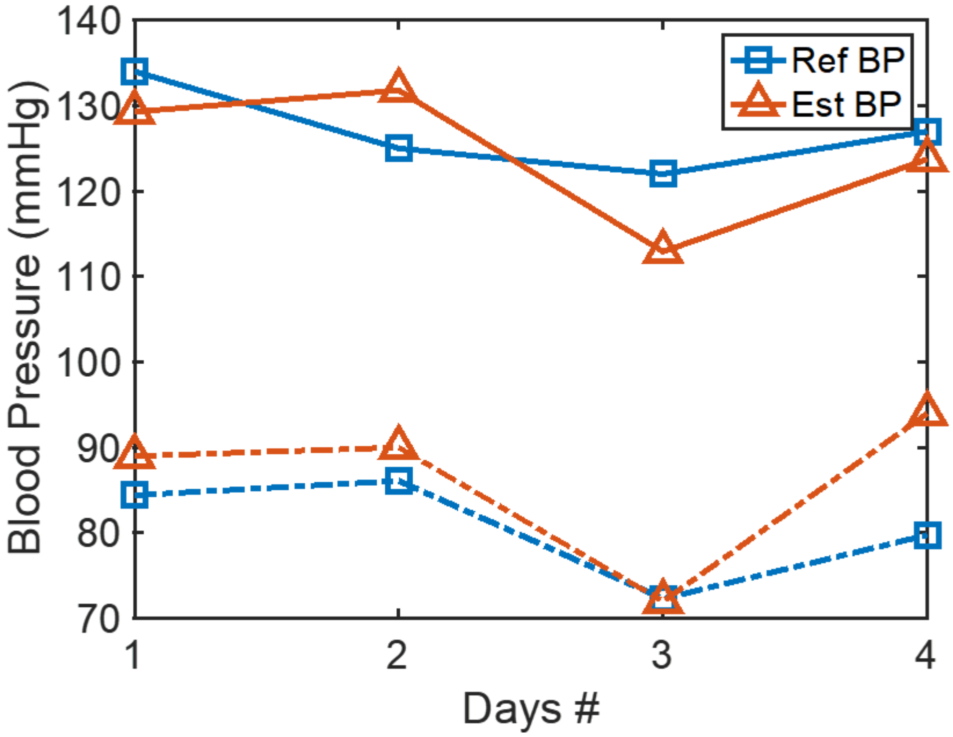

Supplementary Fig. 43. Long-term (4 days) BP tracking of subject 2 through our BP measurement system.

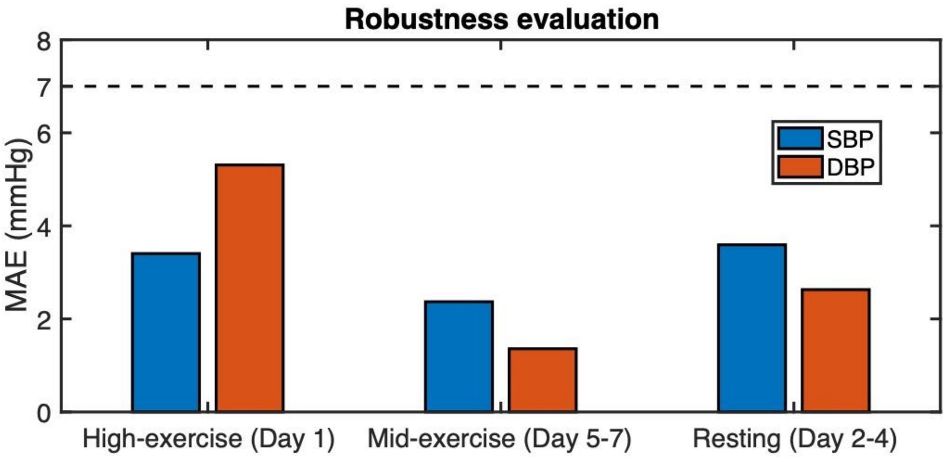

Supplementary Fig. 44. Robustness evaluation of long-term BP tracking.

Supplementary Table 1. Hemodynamic parameters of artery.

	Beta stiffness index (cm^{-2})	Blood density (kg/m^3)
Range	1.43-3.42[15]	1043 -1060 [17]
Relative variation	0.82	0.016

Supplementary Table 2. Estimation error comparison of the proposed dual-modality method with other continuous BP measurement methods.

Modality	Methods*	Elements	Estimation Error														Reference
			MAE(mmHg)		RMSE(mmHg)		m.e.(mmHg)		s.d. (mmHg)		<5 mmHg (%)		<10 mmHg(%)		<15mmHg(%)		
			SBP	DBP	SBP	DBP	SBP	DBP	SBP	DBP	SBP	DBP	SBP	DBP	SBP	DBP	
Single-modality with 1-element model	$P_d e^{\bar{\beta}\left(\frac{D(t)}{D_d}-1\right)}$	*D*	13.62	7.74	17.01	9.10	-6.38	-3.06	15.80	8.59	23.84	31.67	45.91	66.90	62.63	86.83	[18]
	$a_1 \cdot PWV + b_1$	*PWV*	7.77	5.01	10.29	6.17	-2.83	-1.50	9.91	5.99	46.62	59.79	63.35	93.95	85.77	96.80	[19]
	$a_2 \cdot PWV^2 + b_2$	*PWV*	7.52	4.88	10.14	6.04	-2.27	-1.10	9.90	5.95	52.67	60.85	66.55	91.10	86.48	96.80	[20]
Dual-modality with 2-element model	$\frac{PWV^2}{\left[\frac{A(A-A_0)}{A_0^2}\right]} \cdot a_3 + b_3$	*PWV, A*	9.41	5.32	11.69	7.20	3.95	2.94	11.02	6.58	29.89	61.92	61.21	81.14	79.36	93.95	[20]
	$\frac{PWV^2 D_d}{D} \cdot a_4 + b_4$	*PWV, D*	7.17	4.72	9.77	5.83	-1.63	-0.66	9.65	5.80	53.02	62.99	68.68	92.88	88.26	96.80	[21]
Dual-modality with 3-element model	$\frac{PWV^2}{A} \cdot \mu + \theta$	*PWV, A,* μ	**5.22**	**4.57**	**7.18**	**5.78**	**0.90**	**1.77**	**7.14**	**5.51**	**60.33**	**61.15**	**86.36**	**93.80**	**92.56**	**97.10**	**This work**

*a_1 and b_1 are the calibration coefficients of the *PWV*-BP method based on the experiments[19]; a_2 and b_2, in the PWV^2-BP method, depend on the material properties and geometry of the artery and are to be determined from the experiments[20] ; a_3 and b_3 are the calibration coefficients that related to elastic modulus (at zero pressure) and blood density of the PWV^2-*A* to BP method[20]; a_4 is the ratio of blood density and beta stiffness and b_4 is a constant shift between the model-based BP and measured BP of the PWV^2-*Diam* to BP method[21].

Supplementary Table 3. Estimation Error of BP across diverse subpopulations.

Subpopulations		Estimation Error (mmHg)							
		MAE		RMSE		m.e.		s.d.	
		SBP	DBP	SBP	DBP	SBP	DBP	SBP	DBP
Gender	Male	5.34	4.55	7.40	6.04	3.49	1.62	6.55	5.84
	Female	5.07	4.59	7.25	5.49	-3.09	2.64	6.60	4.84
Age	⩾26 years	5.33	4.63	7.32	6.06	2.35	2.52	6.95	5.52
	<26 years	5.01	4.41	7.41	5.22	-2.39	0.58	7.07	5.23
Skin-tone	Medium	5.19	5.06	7.49	6.38	0.68	2.75	7.49	5.78
	Dark	5.43	3.32	6.98	4.11	4.14	0.04	5.69	4.17
	Light	5.36	2.68	6.71	3.11	-1.80	-0.78	6.65	3.10

Supplementary Table 4. Mean error and standard deviation of BP across diverse subpopulations with individual calibration.

Subject	SBP (mmHg)		DBP(mmHg)		Subject	SBP (mmHg)		DBP(mmHg)	
No.	m.e.	s.d.	m.e.	s.d.	No.	m.e.	s.d.	m.e.	s.d.
**1**	0.06	0.84	-2.00	0.53	**24**	6.84	0.61	0.80	0.39
**2**	-1.27	0.86	3.55	0.54	**25**	1.48	0.19	2.12	0.12
**3**	-2.06	0.90	-2.32	0.58	**26**	-6.72	0.94	0.77	0.64
**4**	-0.63	3.05	-2.81	2.00	**27**	-1.16	0.81	-1.24	0.57
**5**	2.47	0.10	1.95	0.07	**28**	0.69	0.60	1.12	0.39
**6**	-5.22	1.51	-3.80	0.95	**29**	-0.28	0.47	2.97	0.30
**7**	-3.28	0.48	-0.38	0.30	**30**	1.33	0.48	2.27	0.34
**8**	-1.90	0.43	0.05	0.27	**31**	-2.76	0.57	-1.03	0.44
**9**	-6.22	0.35	-0.54	0.21	**32**	-1.48	0.43	-0.76	0.29
**10**	-1.03	1.06	2.24	0.67	**33**	3.31	0.08	6.60	0.05
**11**	-4.73	0.44	-0.26	0.27	**34**	4.15	1.60	4.67	1.27
**12**	-10.10	0.22	-3.42	0.14	**35**	1.41	2.20	-1.24	1.70
**13**	-1.23	0.93	-2.30	0.58	**36**	-0.30	0.29	-0.44	0.21
**14**	4.36	1.67	0.19	1.06	**37**	5.03	0.56	-2.58	0.36
**15**	-2.44	1.13	-3.35	0.72	**38**	1.40	0.89	1.93	0.59
**16**	-3.69	1.15	1.48	0.73	**39**	4.07	1.40	-0.93	1.06
**17**	0.15	4.49	-3.44	3.15	**40**	8.39	1.48	5.12	1.14
**18**	1.79	0.38	6.06	0.24	**41**	0.31	0.45	-1.57	0.33
**19**	1.45	0.46	0.99	0.29	**42**	2.50	1.04	2.76	0.79
**20**	-2.36	0.19	-1.47	0.12	**43**	4.54	0.25	-0.39	0.17
**21**	0.09	0.24	-1.39	0.15	**44**	1.62	1.28	0.88	0.90
**22**	0.94	0.73	-0.45	0.45	**45**	4.11	1.22	-0.81	0.89
**23**	-4.20	1.49	0.76	0.95					

Supplementary Table 5. Mean error and standard deviation of BP across diverse subpopulations without calibration.

Subject No.	SBP (mmHg)		DBP(mmHg)		Subject No.	SBP (mmHg)		DBP(mmHg)	
	m.e.	s.d.	m.e.	s.d.		m.e.	s.d.	m.e.	s.d.
**1**	-2.58	2.41	-5.31	1.89	**24**	17.70	1.29	6.66	0.78
**2**	-6.80	2.12	3.90	1.59	**25**	-0.27	0.56	2.25	0.43
**3**	-0.71	1.67	0.35	1.02	**26**	-13.44	1.88	1.54	1.28
**4**	3.78	5.48	-0.11	3.09	**27**	-2.33	1.62	-2.48	1.15
**5**	7.81	0.29	8.53	0.21	**28**	1.38	1.21	2.23	0.78
**6**	-8.46	3.95	-3.67	2.83	**29**	-0.56	0.93	5.94	0.59
**7**	-8.78	1.66	-1.68	1.39	**30**	2.66	0.97	4.53	0.68
**8**	1.08	0.93	4.94	0.52	**31**	-5.52	1.14	-2.07	0.88
**9**	-8.74	0.93	4.19	0.62	**32**	-2.97	0.86	-1.53	0.59
**10**	-1.31	2.97	7.13	2.21	**33**	6.63	0.15	13.19	0.10
**11**	-5.53	1.23	4.93	0.85	**34**	8.29	3.20	9.34	2.54
**12**	-20.85	0.93	-6.04	0.84	**35**	2.81	4.39	-2.49	3.39
**13**	-0.18	2.28	-0.51	1.57	**36**	-0.60	0.57	-0.88	0.42
**14**	1.82	4.01	-6.22	3.07	**37**	10.05	1.13	-5.16	0.73
**15**	-2.71	2.49	-2.72	1.59	**38**	2.79	1.78	3.86	1.17
**16**	-3.26	2.40	8.62	1.11	**39**	8.14	2.81	-1.86	2.12
**17**	9.46	7.24	4.16	4.05	**40**	16.77	2.97	10.24	2.28
**18**	5.77	1.06	16.23	0.77	**41**	0.61	0.90	-3.15	0.66
**19**	2.24	1.15	3.08	0.84	**42**	4.99	2.09	5.53	1.59
**20**	-0.10	0.45	2.34	0.25	**43**	9.07	0.50	-0.78	0.34
**21**	0.66	0.74	-0.41	0.58	**44**	3.25	2.57	1.76	1.80
**22**	3.51	1.95	2.66	1.39	**45**	8.22	2.44	-1.62	1.79
**23**	-3.75	2.97	7.05	1.25					

Supplementary Table 6. Estimation error of 7 days BP tracking of subject 1.

Individual long-term BP tracking		Days #							Avg.
		1	2	3	4	5	6	7	
MAE (mmHg)	SBP	3.4	3.48	3.31	4	2.28	2.54	2.3	3.044
	DBP	5.31	2.53	2.08	3.2	3.68	0.84	0.74	2.625
RMSE (mmHg)	SBP	3.44	4.16	4.25	4.08	2.32	2.55	2.59	3.341
	DBP	5.6	3	2.33	3.7	3.69	0.85	0.94	2.872
m.e. (mmHg)	SBP	2.58	-2.66	-2.29	1.68	-2.28	-2.54	2.3	-0.458
	DBP	5.31	-2.06	0.61	2.75	3.68	0.84	-0.27	1.551
s.d. (mmHg)	SBP	2.41	3.34	4.13	4.08	0.62	0.25	1.33	2.308
	DBP	1.89	2.27	2.6	2.7	0.41	0.18	1.01	1.58

Supplementary Table 7. Estimation error of 4 days BP tracking of subject 2.

Individual long-term BP tracking		Days #				Avg.
		1	2	3	4	
MAE (mmHg)	SBP	4.72	6.80	9.07	3.27	5.96
	DBP	4.57	3.90	0.31	14.23	5.75
RMSE (mmHg)	SBP	5.32	7.06	9.08	3.37	6.35
	DBP	5	4.15	0.40	14.24	7.72
m.e. (mmHg)	SBP	-4.72	6.80	-9.07	-3.27	-2.56
	DBP	-4.57	-3.90	0.31	-14.23	-5.59
s.d. (mmHg)	SBP	2.82	2.12	0.50	1	6.71
	DBP	2.33	1.59	0.35	0.74	6.14

Supplementary Table 8. Computational complexity comparison of the proposed dual-modality method with other continuous BP measurement methods.

Methods	Time complexity	Space complexity	Comments	Degree of Complexity	Accuracy* in population	Calibration-free?
$a_1 \cdot PWV + b_1$	**Training：O(1)<br>Test：O(N)**	**Parameter storage: O(1)<br>Output storage-N samples: O(N)**	**1 multiplication (a ×PTT) and 1 addition (+b)**	**low**	**low**	**No**
$a_2 \cdot PWV^2 + b_2$	**Training：O(1)<br>Test：O(N)**	**Parameter storage: O(1)<br>Output storage-N samples: O(N)**	**2 multiplications + 1 addition**	**low**	**low**	**No**
$P_d e^{\beta\left(\frac{D(t)}{D_d}-1\right)}$	**Training: O(K×N)（K iteration number）<br>Test：O(N)**	**Parameter storage: O(1)<br>Output storage-N samples: O(N)**	**1 division + 2 multiplication + 1 subtraction + 1 exponential operation + 1 addition**	**median**	**low**	**No**
$\frac{PWV^2}{\left[\frac{A(A-A_0)}{A_0^2}\right]} \cdot a_4 + b_4$	**Training：O(K ×N)（K iteration number）<br>Test：O(N)**	**Parameter storage: O(1)<br>Output storage-N samples: O(N)**	**Multiplication: 3 times (with PWV^2, A ×(A-A$_0$), ×a)<br>Division: 2 times<br>Addition/subtraction: 1 time each**	**median**	**low**	**No**
$\frac{PWV^2 D_d}{D} \cdot a_3 + b_3$	**Training：O(K ×N)（K iteration number）<br>Test：O(N)**	**Parameter storage: O(1)<br>Output storage-N samples: O(N)**	**2 multiplications + 1 division + 1 addition**	**median**	**low**	**No**
$\frac{PWV^2}{A} \cdot \mu + \theta$	**Training：O(K ×N)（K iteration number）<br>Test：O(N)**	**Parameter storage: O(1)<br>Output storage-N samples: O(N)**	**4 multiplications + 3 divisions + 2 additions + 2 subtractions + 1 logarithmic number**	**median**	**high**	**Yes**

* The detailed accuracy comparison is provided in Supplementary Table 2.

O(1): constant complexity

O(N): linear complexity

O(K×N): quadratic complexity

Supplementary Table 9. Technical comparison of other similar BP monitoring devices.

Method	Cuffless (yes or not)	Modalities for BP measurement	Continuous (yes or not)	Accuracy				Long-term tracking without calibration?	Population disparities without calibration?
				SBP (mmHg)		DBP (mmHg)			
				m.e.	s.d.	m.e.	s.d.		
PPG[22]	Not	1, PTT	Not	2.12	0.26	2.94	0.72	Not	Not
Bio-Z[12]	Yes	1, PTT	Yes	0.2	5.8	0.2	4.5	Not	Not
Pressure sensor[8]	Not	1, PWV	Yes	-0.05	4.61	0.11	3.68	Not	Not
Epidermal Pressure sensor[23]	Yes	1, pressure	Yes	−0.89	6.19	−0.32	5.28	Not	Not
Ultrasound Transducer[18]	Yes	1, Diameter	Yes	-1.85	1.52	-4.01	0.85	Not	Not
Ultrasound Transducer[24]	Yes	1, Diameter	Yes	0.17 ± 1.96				Not	Not
Ultrasound Transducer[13]	Yes	1, Diameter	Yes	0.34	3.90	-0.43	2.66	Yes	Not
**Ultrasound transducer + pressure sensor★**	**Yes**	**2, Diameter and PWV**	**Yes**	**0.52#**	**3.38#**	**0.88#**	**2.75#**	**Yes**	**Yes**
				**0.90***	**7.14***	**1.77***	**5.51***		

★: This work

#: Results of different populations with recalibration

*: Results of different populations without recalibration

Reference

[1] Mukkamala, R., Stergiou, G. S. & Avolio, A. P. Cuffless blood pressure measurement. *Annu. Rev. Biomed. Eng.* **24**, 203-230 (2022).

[2] Pandit, J. A., Lores, E. and Batlle, D. Cuffless blood pressure monitoring: promises and challenges. *Clin. J. Am. Soc. Nephrol.* **15**, 1531-1538 (2020).

[3] Luo, N. et al. Flexible piezoresistive sensor patch enabling ultralow power cuffless blood pressure measurement. *Adv. Funct. Mater.* **26**, 1178-1187 (2016).

[4] Panula, T. et al. Advances in non-invasive blood pressure measurement techniques. *IEEE Rev. Biomed. Eng.* **16**,424-438(2022).

[5] Chung, E. et al. Non-invasive continuous blood pressure monitoring: a review of current applications. *Front. Med.* **7**, 91-101 (2013).

[6] Kumar, N. V. et al. Fiber bragg grating-based pulse monitoring device for real-time non-invasive blood pressure measurement-a feasibility study. *IEEE Sens. J.* **21**, 9179-9185 (2021).

[7] Kim, J. et al. Soft wearable pressure sensors for beat-to-beat blood pressure monitoring. *Adv. Healthc. Mater.* **8**, 1900109 (2019).

[8] Li, J. et al. Thin, soft, wearable system for continuous wireless monitoring of artery blood pressure. *Nat. Commun.* **14**, 5009 (2023).

[9] Liu, C. et al. Wireless, skin-interfaced devices for pediatric critical care: application to continuous, noninvasive blood pressure monitoring. *Adv. Healthc. Mater.* **10**, 2100383 (2021).

[10] Liu, J. et al. Multi-wavelength photoplethysmography enabling continuous blood pressure measurement with compact wearable electronics. *IEEE Trans. Biomed. Eng.* **66**, 1514-1525 (2018).

[11] Hernández-Urrea, M. et al. An easy-to-use hand-to-hand impedance-based sensor to obtain carotid pulse arrival time. *IEEE Sens. J.* **23**, 5362-5369 (2023).

[12] Kireev, D. et al. Continuous cuffless monitoring of arterial blood pressure via graphene bioimpedance tattoos. *Nat. Nanotechnol.* **17**, 864-870 (2022).

[13] Zhou, S. et al. Clinical validation of a wearable ultrasound sensor of blood pressure. *Nat. Biomed. Eng* **9**, 865-881 (2025).

[14] Jimenez, R. et al. Resonance sonomanometry for noninvasive, continuous monitoring of blood pressure. *PNAS nexus* **3**, pgae252 (2023).

[15] Gavish, B. & Izzo Jr, J. L. Arterial stiffness: going a step beyond. *Am. J. Hypertens.* **29**, 1223-1233 (2016).

[16] Li, Y. et al. Ambulatory arterial stiffness index derived from 24-hour ambulatory blood pressure monitoring. *Hypertension* **47**, 359-364 (2006).

[17] Johar, R. S. & Smith, R. P. Assessing gravimetric estimation of intraoperative blood loss. *J. Gynecol. Surg.* **9**, 151-154 (1993).

[18] Wang, C. et al. Monitoring of the central blood pressure waveform via a conformal ultrasonic device. *Nat. Biomed. Eng* **2**, 687-695 (2018).

[19] Young, C. C. et al. Clinical evaluation of continuous noninvasive blood pressure monitoring: accuracy and tracking capabilities. *J. Clin. Monit.* **11**, 245-252 (1995).

[20] Ma, Y. et al. Relation between blood pressure and pulse wave velocity for human arteries. *Proc. Natl. Acad. Sci. U. S. A.* **115**, 11144-11149 (2018).

[21] Guo, C. Y. et al. Combining local PWV and quantified arterial changes for calibration-free cuffless blood pressure estimation: A clinical validation. *IEEE Sens. J.* **23**, 658-668 (2022).

[22] Byfield, R. et al. Towards robust blood pressure estimation from pulse wave velocity measured by photoplethysmography sensors. *IEEE Sens. J.* 22, 2475-2483 (2021).

[23] Min, S. et al. Clinical validation of a wearable piezoelectric blood-pressure sensor for continuous health monitoring. *Adv. Mater.* **35**, 2301627 (2023).

[24] Lin, M. et al. A fully integrated wearable ultrasound system to monitor deep tissues in moving subjects. *Nat. Biotechnol.* **42**, 448-457 (2024).

Supplementary Movie 1. Measurement process of vascular diameter and PWV using the CSPM
