# Supplementary material for: A conformal piezoelectric microsystem for demographic-adaptive and calibration-free cuffless blood pressure monitoring
Source: Nat Commun. 2025 Dec 9;17:439. doi: 10.1038/s41467-025-67118-4 (PMC12799606; doi:10.1038/s41467-025-67118-4)
Supplement: Supplementary file 2 — Description Of Additional Supplementary File [file 41467_2025_67118_MOESM2_ESM.pdf]

Description of Additional supplementary files

Supplementary Movie 1. Measurement process of vascular diameter and PWV using the CSPM
